# RqcH and RqcP catalyze processive poly-alanine synthesis in a reconstituted ribosome-associated quality control system

**DOI:** 10.1093/nar/gkab589

**Published:** 2021-07-13

**Authors:** Hiraku Takada, Caillan Crowe-McAuliffe, Christine Polte, Zhanna Yu Sidorova, Victoriia Murina, Gemma C Atkinson, Andrey L Konevega, Zoya Ignatova, Daniel N Wilson, Vasili Hauryliuk

**Affiliations:** Faculty of Life Sciences, Kyoto Sangyo University, Kamigamo, Motoyama, Kita-ku, Kyoto 603-8555, Japan; Department of Molecular Biology, Umeå University, 90187 Umeå, Sweden; Laboratory for Molecular Infection Medicine Sweden (MIMS), Umeå University, 90187 Umeå, Sweden; Institute for Biochemistry and Molecular Biology, University of Hamburg, 20146 Hamburg, Germany; Institute for Biochemistry and Molecular Biology, University of Hamburg, 20146 Hamburg, Germany; Petersburg Nuclear Physics Institute named by B.P. Konstantinov of National Research Centre “Kurchatov Institute”, 188300 Gatchina, Russia; Russian Research Institute of Hematology and Transfusiology of FMBA, 191024 Saint Petersburg, Russia; Department of Molecular Biology, Umeå University, 90187 Umeå, Sweden; Laboratory for Molecular Infection Medicine Sweden (MIMS), Umeå University, 90187 Umeå, Sweden; National Research Centre “Kurchatov Institute”, 123182 Moscow, Russia; Petersburg Nuclear Physics Institute named by B.P. Konstantinov of National Research Centre “Kurchatov Institute”, 188300 Gatchina, Russia; Peter the Great St. Petersburg Polytechnic University, 195251 Saint Petersburg, Russia; National Research Centre “Kurchatov Institute”, 123182 Moscow, Russia; Institute for Biochemistry and Molecular Biology, University of Hamburg, 20146 Hamburg, Germany; Institute for Biochemistry and Molecular Biology, University of Hamburg, 20146 Hamburg, Germany; Department of Molecular Biology, Umeå University, 90187 Umeå, Sweden; Laboratory for Molecular Infection Medicine Sweden (MIMS), Umeå University, 90187 Umeå, Sweden; Department of Experimental Medical Science, Lund University, 221 00 Lund, Sweden; University of Tartu, Institute of Technology, 50411 Tartu, Estonia

## Abstract

In the cell, stalled ribosomes are rescued through ribosome-associated protein quality-control (RQC) pathways. After splitting of the stalled ribosome, a C-terminal polyalanine ‘tail’ is added to the unfinished polypeptide attached to the tRNA on the 50S ribosomal subunit. In *Bacillus subtilis*, polyalanine tailing is catalyzed by the NEMF family protein RqcH, in cooperation with RqcP. However, the mechanistic details of this process remain unclear. Here we demonstrate that RqcH is responsible for tRNA^Ala^ selection during RQC elongation, whereas RqcP lacks any tRNA specificity. The ribosomal protein uL11 is crucial for RqcH, but not RqcP, recruitment to the 50S subunit, and *B. subtilis* lacking uL11 are RQC-deficient. Through mutational mapping, we identify critical residues within RqcH and RqcP that are important for interaction with the P-site tRNA and/or the 50S subunit. Additionally, we have reconstituted polyalanine-tailing *in vitro* and can demonstrate that RqcH and RqcP are necessary and sufficient for processivity in a minimal system. Moreover, the *in vitro* reconstituted system recapitulates our *in vivo* findings by reproducing the importance of conserved residues of RqcH and RqcP for functionality. Collectively, our findings provide mechanistic insight into the role of RqcH and RqcP in the bacterial RQC pathway.

## INTRODUCTION

In all cells, the process of synthesizing proteins by ‘reading’ the coded instructions in mRNA is called translation, and it is carried out by the molecular machine called the ribosome. Damaged or truncated mRNAs are harmful to cells because they sequester ribosomes from active protein production and can result in the synthesis of cytotoxic truncated proteins (1). Therefore, diverse ribosome rescue pathways have evolved in all domains of life to disassemble stalled ribosomal complexes (2–4). The first ribosome rescue pathway to be identified in bacteria was the trans-translation system (5). The system consists of transfer-messenger RNA (tmRNA; also referred to as small stable RNA A, *ssrA*) that serves both as an mRNA template (6) and a tRNA mimic (7). This RNA component of tmRNA is assisted by small protein B (SmpB) (8). Acting as a tRNA, alanyl-tmRNA mediates the addition of alanine to ribosome-stalled nascent polypeptide chains (7). Next, decoding of the ORF encoded within the tmRNA results in the addition of the short C-terminal ssrA peptide tag (6). This, in turn, marks the aberrant proteins for degradation by ClpA, ClpXP and FtsH proteases (9). In addition to the trans-translation system, *Escherichia coli* possess two alternative rescue systems that are related to or rely on the canonical stop codon-dependent translation termination machinery involving class-1 release factors RF1 and RF2 (2,3). While alternative rescue factor A (ArfA) recruits RF2 to release the stalled polypeptide in the absence of the A-site stop codon (10), ArfB acts as a peptidyl hydrolase itself (11). Unlike the trans-translation system, which is universally conserved in bacteria (12,13), ArfA and ArfB have patchy evolutionary distributions, and are missing, for example, in the model firmicute bacterium *Bacillus subtilis* or the human pathogen *Francisella tularensis* (14). Instead, these species rely on release factor-dependent alternative rescue systems ArfT (*F. tularensis* (15)) and BrfA (*B. subtilis* (16)).

The translation apparatus of both archaea and the eukaryotic cytoplasm lack the trans-translation and Arf/Brf ribosome rescue systems. Instead, they rely on ribosome-associated protein quality control, RQC (17,18). RQC is executed by the concerted action of Ribosome quality control complex subunits 1 (Rqc1) and 2 (Rqc2, aka NEMF/SDCCAG1 in humans, Caliban in Drosophila and Tae2 in yeast), E3 ubiquitin ligase Ltn1/Listerin and the AAA ATPase Cdc48/p97 (19,20). Unlike the bacterial systems described above that operate on the 70S ribosomes, eukaryotic RQC operates on the large 60S subunit (19,20) which can be generated by splitting the stalled ribosome by Dom34/Pelota-Hbs1 and ABCE1/Rli1 (21–25) or, alternatively, the RQC-trigger/ASC-1 complex (26,27). In yeast, the resulting 60S-P-tRNA complex is a substrate for the mRNA-independent addition of C-terminal extensions of poly-alanine co-polymerised with threonine (CAT tails) (28). CAT tailing progresses the stalled nascent peptide through the exit tunnel, presenting lysine residues in the nascent peptide that are substrates for ubiquitinylation by Ltn1 (29). Off the ribosome, CAT tails promote protein aggregation (30,31) and serve as a degron tag promoting degradation by the proteasome (32). Recently, it has been shown that C-terminal tails mediated by the RQC machinery in mammalian cells and *Drosophila melanogaster* are composed predominantly of alanine, with minor contribution from other amino acids (33–35).

**Figure 1.**
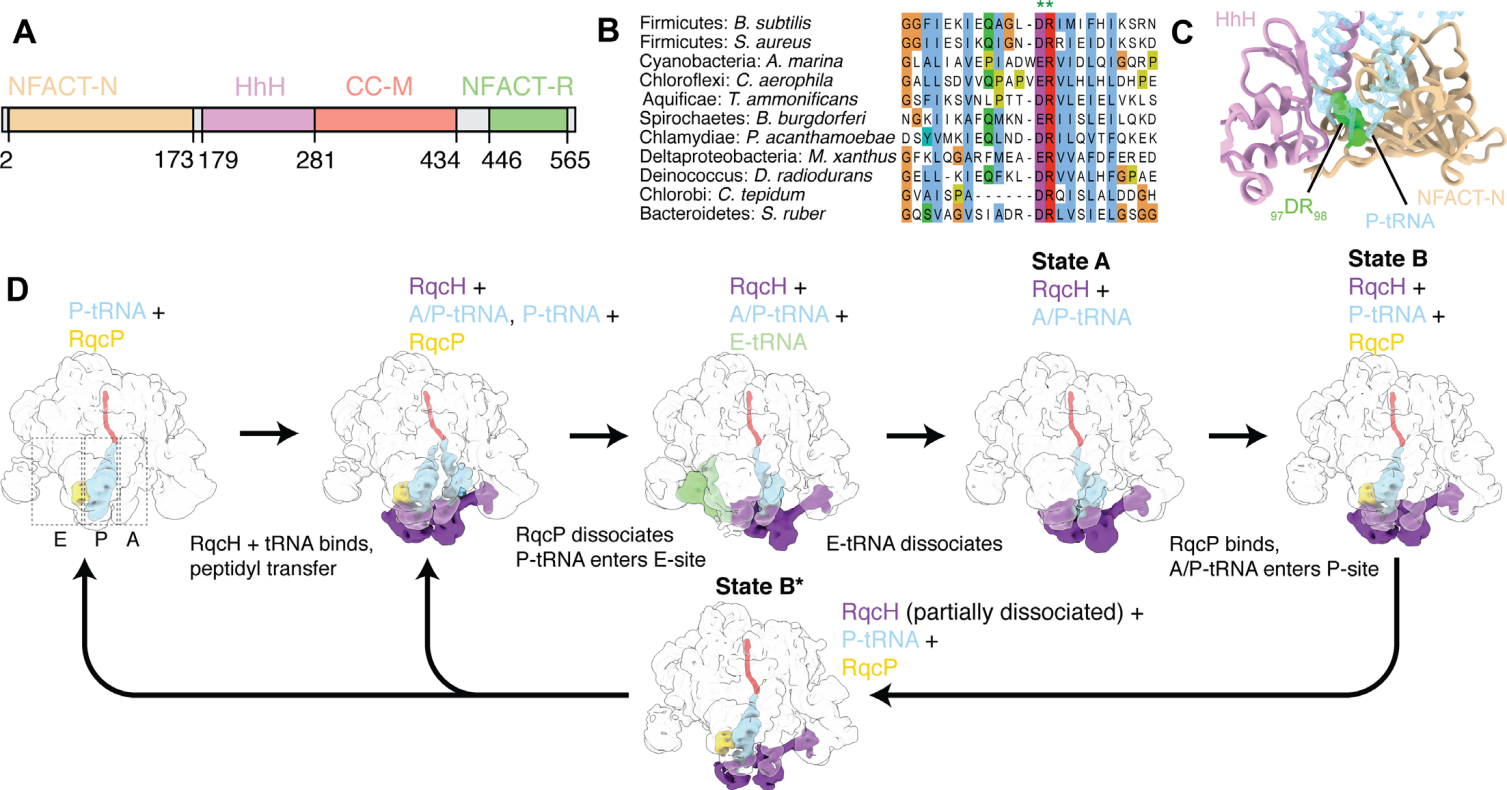
Bacterial ribosome-associated RQC is mediated by RqcH and RqcP. (**A**) RqcH domain composition. Amino acid positions are numbered as per *B. subtilis* RqcH. (**B**) Sequence alignment of RqcH homologs from diverse bacteria. (**C**) Interaction between RqcH (HhH domain in lilac, NFACT-N domain in tan, _97_DR_98_ motif in green) and P-tRNA (transparent cyan) in state B (PDB 7ASA (42)). (**D**) Overview of proposed RQC elongation cycle mediated by RqcH and RqcP (42). Beginning with a 50S with trapped P-tRNA, binding and dissociation cycles of RqcH and RqcP, accompanied by movement of tRNAs through the A-, P- and E-sites, results in mRNA- and 30S-independent elongation.

Diverse bacterial species of phyla across the bacterial tree of life encode NEMF-family fibronectin-binding protein A (FpbA) proteins homologous to eukaryotic Rqc2 proteins (36). For decades, the exact molecular function of these proteins was unclear. *Enterococcus faecalis* EfbA (37), *Listeria monocytogenes* FbpA (38,39) and *Streptococcus pneumoniae* PavA (40) were originally recognized as virulence factors. The situation changed in 2019 when *B. subtilis* FpbA homologue YloA was demonstrated to be a functional analogue as well as a homologue of eukaryotic Rqc2, which led to renaming of the protein to bacterial Rqc2 homolog, RqcH (41). RqcH, like other NEMF family proteins, contains a conserved core domain architecture of NFACT-N (NFACT means domain found in NEMF, FbpA, Caliban, and Tae2), helix-hairpin-helix (HhH), coiled coils (CC), a small β-hairpin-containing middle (M)-domain between the two helices of the CC, and an NFACT-R domain (28,36,41–43) (Figure 1A). RqcH recruits tRNA^Ala^ to 50S-peptidyl-tRNA complexes generated by an as yet unknown molecular players that split stalled 70S ribosomes, and drives the addition of C-terminal poly-alanine tails that, in turn, act as degron tags recognized by the ClpP protease (41). The *rqcH* gene genetically interacts with *ssrA*, as the double deletion mutant *ssrA* and *rqcH* displays an increased sensitivity to antibiotics that inhibit translation and displays a synthetic growth defect at 45°C (41). This genetic interaction suggests that the two ribosome rescue systems have complementary functions in *B. subtilis*.

Cryo-EM structures of RqcH-50S complexes isolated using *in vivo* pull-down approaches have established that the NFACT-N domain of RqcH is the key structural element mediating tRNA^Ala^ recognition (42,44), thus rationalizing the earlier report of D97A/R98A substitution (RqcH^DR^) in the domain ablating RQC functionality (41). Indeed, RqcH appears to interact with both tRNA^Ala^ isoacceptors, namely tRNA^Ala(UGC)^ and tRNA^Ala(GGC)^ (42) (note that the latter was annotated as tRNA^Ala(IGC)^ in the original report due to the hybridization with the microarray probe designated as Ala-IGC, Supplementary Table S1)—and the conserved _97_DR_98_ residues have been suggested to contribute to this specificity by interacting with the anticodon–stem–loop of these tRNAs, in particular the G35 at the central position of the anticodon (42,44) (Figure 1B,C). A similar variant in yeast Rqc2 could bind to 50S-P-tRNA complexes, but not support CAT tailing (28). The structures also revealed that the M domain of RqcH is tethered to ribosomal protein uL11 located at the L7/L12 stalk base, suggesting that this interaction is critical for RqcH function. Furthermore, analysis of the RqcH-50S RQC pull-down complexes led to the discovery of RqcP, an Hsp15-family protein (42,44). By analysing the ensemble of generated cryo-EM structures, a putative mechanism of RqcH/RqcP-driven poly-alanine synthesis on the large 50S subunit was proposed (Figure 1D) (42). By associating with 50S-peptidyl-tRNA complexes, RqcP stabilizes the peptidyl-tRNA in the P-site, thus allowing efficient recruitment of RqcH:tRNA^Ala^ to the A-site. Consequent spontaneous transpeptidation and RqcP dissociation allow the 50S-bound tRNAs to sample different ribosomal binding sites. This results in departure of the deacylated E-site tRNA and allows the peptidyl-tRNA extended with C-terminal alanine residue to adopt a hybrid A/P-like state. Rebinding of RqcP stabilizes the peptidyl-tRNA in the P-site, resulting in a complex with P-site tRNA, RqcH and RqcP. This latter complex was the best-resolved state observed in cryo-EM reconstructions, which we termed state B. An additional state, which was similar to state B, but with partially dissociated RqcH, was termed state B* and assumed to represent a transition towards RqcH dissociation.

Here, we employ biochemical and structural techniques to dissect the role of RqcH and RqcP during bacterial RQC. We show that RqcH can specifically select tRNA^Ala^ in the absence of the 50S subunit and that RqcP does not contribute to this selection specificity. Indeed, we show that mutation of the conserved DR motif to AA leads to loss of this tRNA^Ala^ specificity, supporting the importance of the motif for distinguishing tRNA^Ala^ from other tRNA species. A cryo-EM structure of a RqcH^DR^ mutant in complex with the peptidyl–tRNA–50S complex reveals that the NFACT-N domain of the RqcH^DR^ mutant has disengaged from the anticodon stem loop of the P-site tRNA, thus providing a structural basis for the loss in specificity. In addition, we demonstrate that uL11 is critical for RqcH recruitment to the 50S subunit and *B. subtilis* lacking uL11 is RQC-deficient. We also identify critical residues within RqcH and RqcP that are important for recruitment to the peptidyl-tRNA 50S complexes. Furthermore, we establish an *in vitro*-reconstituted polyalanine-tailing assay, which we use to demonstrate that RqcH and RqcP are necessary and sufficient for processivity of polyalanine tailing. The *in vitro*-reconstituted polyalanine-tailing assay opens the way for further studies to dissect the role of other factors involved in bacterial RQC.

## MATERIALS AND METHODS

Strains, plasmids (and details regarding the construction thereof) as well as oligonucleotides and synthetic DNA sequences used in this study are provided in Supplementary Table S2. Detailed description of protein and tRNA purification procedures is provided in the *Supplementary methods*.

### Multiple sequence alignment

RqcH and RqcP sequences were extracted from the previously reported dataset (42), aligned with MAFFT v7.164b with the L-ins-i strategy (45) and alignments were visualized with Jalview (46).

### Preparation of *B. subtilis* 50S ribosomal subunits

*B. subtilis* 70S was purified as described earlier (47), diluted with low-magnesium HEPES:Polymix buffer (1 mM MgOAc) and incubated on ice for 30 minutes to promote the dissociation of subunits. The sample was then resolved on a 10–40% sucrose gradient in overlay buffer (60 mM NH_4_Cl, 1 mM Mg(OAc)_2_, 0.25 mM EDTA, 3 mM β-mercaptoethanol, 20 mM Tris–HCl pH 7.5) in a zonal rotor (Ti 15, Beckman, 18 h at 21 000 rpm). The peak containing pure 50S subunits was pelleted by centrifugation (48 h at 35 000 rpm), and the final ribosomal preparation was dissolved in HEPES:Polymix buffer with 5 mM MgOAc.

### Growth assays

*B. subtilis 168* wild-type and deletion strains were pre-grown on LB plates overnight at 30°C. Fresh individual colonies were used to inoculate liquid LB medium cultures (OD_600_ adjusted to 0.01) at 37°C. Log phase cultures (OD_600_ of ≈0.4) diluted to OD_600_ of 0.1 were used to prepare 10- to 10^5^-fold serial dilutions which were then spotted onto LB agar plates with or without 1 mM IPTG. The plates were scored after 18 hours incubation at either 37°C or 49°C.

### Immunoprecipitation of FLAG_3_-tagged proteins

The experiments were performed as described earlier (42). Strains expressing FLAG_3_-tagged proteins were pre-grown on LB plates overnight at 30°C. Fresh individual colonies were used for inoculation and grown in LB medium. 3× 1 liter cultures were grown at 37°C to OD_600_ = 0.8. Cells were collected by centrifugation (8000 rpm for 10 min at 4°C, JLA-16.25 Beckman Coulter rotor), pellets frozen in liquid nitrogen and stored at –80°C. Cell pellets were resuspended in 8 ml of cell opening buffer (95 mM KCl, 5 mM NH_4_Cl, 20 mM HEPES (pH 7.5), 1 mM DTT, 15 mM Mg(OAc)_2_, 0.5 mM CaCl_2_, 8 mM putrescine, 1 mM spermidine, 1 tablet of cOmplete™ EDTA-free Protease Inhibitor Cocktail (Roche) per 50 ml of buffer) and disrupted using FastPrep homogeniser (MP Biomedicals) with 0.1 mm Zirconium beads (Techtum) in 6 cycles by 20 s with 3 min chill on ice. Cell debris was removed by centrifugation at 14 800 rpm for 20 min 4°C in F241.5P rotor using a 149 Microfuge 22R centrifuge (Beckman Coulter). The supernatant was combined with 100 μl of ANTI-FLAG M2 Affinity Gel (Sigma) pre-equilibrated in cell opening buffer, and incubated for 1.5 h at 4°C on a turning wheel (Fisherbrand™ Multi-Purpose Tube Rotators). The samples were loaded on Micro Bio-Spin columns (Bio-Rad) pre-equilibrated in cell opening buffer, and washed 10 times with 1 ml of cell opening buffer by gravity flow. RqcH-FLAG_3_ was eluted by addition of 200 μl opening buffer containing 0.1 mg/ml poly-FLAG peptide (Biotool, Bimake) for 45 min on a turning wheel. All incubations, washes and elutions were performed at 4°C. The eluted sample was collected by centrifugation at 2000 rpm for 1 min 4°C in a F241.5P rotor using a 149 Microfuge 22R centrifuge (Beckman Coulter). One aliquot of the eluted sample was resolved on SDS-PAGE, the other was blotted on cryo-EM grids, and the remaining sample was used for tRNA-array analyses. For SDS-PAGE analyses, 20 μl aliquots of samples were mixed with 5 μL of 5× SDS loading buffer and heated at 95°C for 15 min, and denatured samples were loaded on 12% SDS-PAGE. SDS-gels were stained by ‘Blue-Silver’ Coomassie Staining (48) and washed with water for 6 h or overnight before imaging with LAS4000 (GE Healthcare).

### Preparation of cryo-EM grids

Eluted pull-down samples were kept on ice and loaded on grids within 2 h after preparation without freezing. The concentration of ribosomes in the samples was estimated from SDS-PAGE gels by comparison of ribosomal band intensities in eluted samples with the bands from loaded ribosomes with known concentration (Supplementary Figure S1). The concentration of ribosomes in elution of RqcH^DR^-FLAG_3_ was approximately 50 nM. Samples were loaded on Quantifoil 2/1 Cu300 grids three times with manual blotting followed by the final blotting using the Vitrobot (FEI). Vitrobot blotting was performed at 100% humidity, 4°C, 5 s blot time, 1 s wait time and 0 s drain time; the resultant sample was vitrified by plunge-freezing in liquid ethane. Grids were imaged on a Titan Krios (FEI) operated at 300 kV at a nominal magnification of 165 000× and a pixel size of 0.82 Å with a Gatan K2 Summit camera with a 4 s exposure and 20 frames using the EPU software.

### Cryo-EM data analysis

Unless otherwise specified, all processing was performed in RELION 3.1 (49). 2510 raw micrograph stacks were motion-corrected with MotionCor2 (50) and the CTF was estimated with CTFFIND4 (51) (Supplementary Figure S2, Table S3). Micrographs with an estimated CTF fit that was significant beyond 4 Å were selected for further processing. crYOLO was used to pick particles (52), resulting in 145 631 initial particles which were extracted with 3× down-sampling. After 2D classification, the remaining 125 573 particles were used to create an *ab initio* model, which was subsequently used as a reference for 3D refinement and 3D classification without angular sampling (53). The majority of particles were grouped into a single class containing a 50S ribosomal large subunit, P-tRNA, RqcP and weak surrounding density. This class was selected for another 3D refinement, followed by partial signal subtraction with a mask around the A, P and E sites (54). Another 3D classification was then performed without angular sampling, the regularization parameter T set to 50, and the resolution used in the expectation step limited to 7 Å. Classes of interest were re-extracted with the original pixel size, refined, and post-processed. FSCs were assessed using the ‘gold-standard’ approach (55). Local resolution was estimated with ResMap (56). For analysis, parts of an existing model of a bacterial RQC complex (PDB: 7AS8) were fitted, by domain, into density using ChimeraX (57).

### Electrophoretic mobility shift assay (EMSA)

Experiments were performed as described earlier (58). Before performing the experiment, stock mRNA(MVF) (5′-GGC**AAGGAGGA**GAUAAGAAUGGUUUUCUAAUA-3′) was incubated for 2 min at 60°C to denature possible secondary structures. Reaction mixtures (10 μL) in HEPES:Polymix buffer (47) with 5 mM Mg^2+^ were assembled by adding either *E. coli* tRNA^Val^ or *B. subtilis* tRNA^Ala^ or tRNA^Lys^ (0.1 μM final concentration) as well as mRNA mRNA(MVF) competitor (1 μM final concentration), followed by the addition of RqcH-HTF (either wild-type or D97A R98A substituted). After incubation for 5 min at 37°C, 4 μL of 50% sucrose was added per sample, and the samples were electrophoretically resolved on a 12% Tris:borate:EDTA gel at 4°C (160–180 V) for 1–1.5 h. Gels were stained with SYBR Gold nucleic acid stain (Life Technologies) for 30 min, followed by visualization using a Typhoon Trio Variable Mode Imager (Amersham Biosciences). Bands were quantified using ImageJ (59). The efficiency of complex formation (effective concentration, EC_50_ ± standard deviation) was calculated using the 4PL model (Hill equation) as per Sebaugh (60) using eight data points; each experiment was performed at least two times.

### Immunoprecipitation of purified His_6_-TEV-FLAG_3_-tagged RqcH variants with *B. subtilis* total tRNA

1 μM purified His_6_-TEV-FLAG_3_-tagged RqcH variants (wild-type and D97A/R98A) and 10 μM *B. subtilis* total tRNA were mixed on ice in IP buffer (HEPES:Polymix buffer pH 7.5, 1 mM DTT, 5 mM MgOAc; 200 μl total volume). The sample was mixed with 50 μl of ANTI-FLAG M2 Affinity Gel (Sigma) pre-equilibrated in IP buffer, and incubated for 0.5 h at 4°C on a turning wheel (Fisherbrand™ Multi-Purpose Tube Rotators). The samples were loaded on Micro Bio-Spin columns (Bio-Rad) pre-equilibrated in IP buffer, and washed 4 times with 0.5 ml of IP buffer by gravity flow. RqcH-His_6_-TEV-FLAG_3_ was eluted by addition of 200 μl IP buffer containing 0.1 mg/ml poly-FLAG peptide (Biotool, Bimake) for 30 min on a turning wheel. All incubations, washes and elutions were performed at 4°C. The eluted sample was collected by centrifugation at 2000 rpm for 1 min 4°C in a F241.5P rotor using a 149 Microfuge 22R centrifuge (Beckman Coulter). RNA was extracted twice with 5:1 acidic phenol:chloroform, precipitated with ethanol, and resuspended in ddH_2_O. Samples containing 150 ng of total RNA were separated on 8 M urea-containing 8% polyacrylamide gels. The gel was stained with SYBR Gold (Life technologies) for 30 min, followed by visualization using a Typhoon Trio Variable Mode Imager (Amersham Biosciences).

### tRNA Northern blotting

For deacylation immunoprecipitated samples were incubated with 125 mM Tris–HCl, pH 9.0, 0.1 M EDTA, 0.5% (w/v) SDS at room temperature for 45 min, before neutralization with an equal volume of 1 M NaOAc, pH 5.5. RNA was extracted twice with 5:1 acidic phenol:chloroform, precipitated with ethanol, and resuspended in ddH_2_O. Samples containing 150 ng of total RNA were separated on 8M urea-containing 8% polyacrylamide gels followed by electroblotting to Zeta-probe membranes (Bio-Rad). The blots were sequentially probed for *B. subtilis* tRNA^Ala^, *B. subtilis* tRNA^Lys^ and *E. coli* tRNA^Val^ using ^32^P-labeled oligonucleotides (Supplementary Table S2). The probes for *B. subtilis* tRNA^Ala^ were designed to hybridize with the conserved TΨC-loop of both tRNA^Ala^ isoacceptors. Signals were detected by phosphorimaging using a Typhoon FLA 9500 biomolecular imager. In parallel, a second gel was subjected to SYBR Gold (Life Technologies) nucleic acid staining for 30 min, followed by visualization using a Typhoon Trio Variable Mode Imager (Amersham Biosciences).

### tRNA microarrays

**Figure 2.**
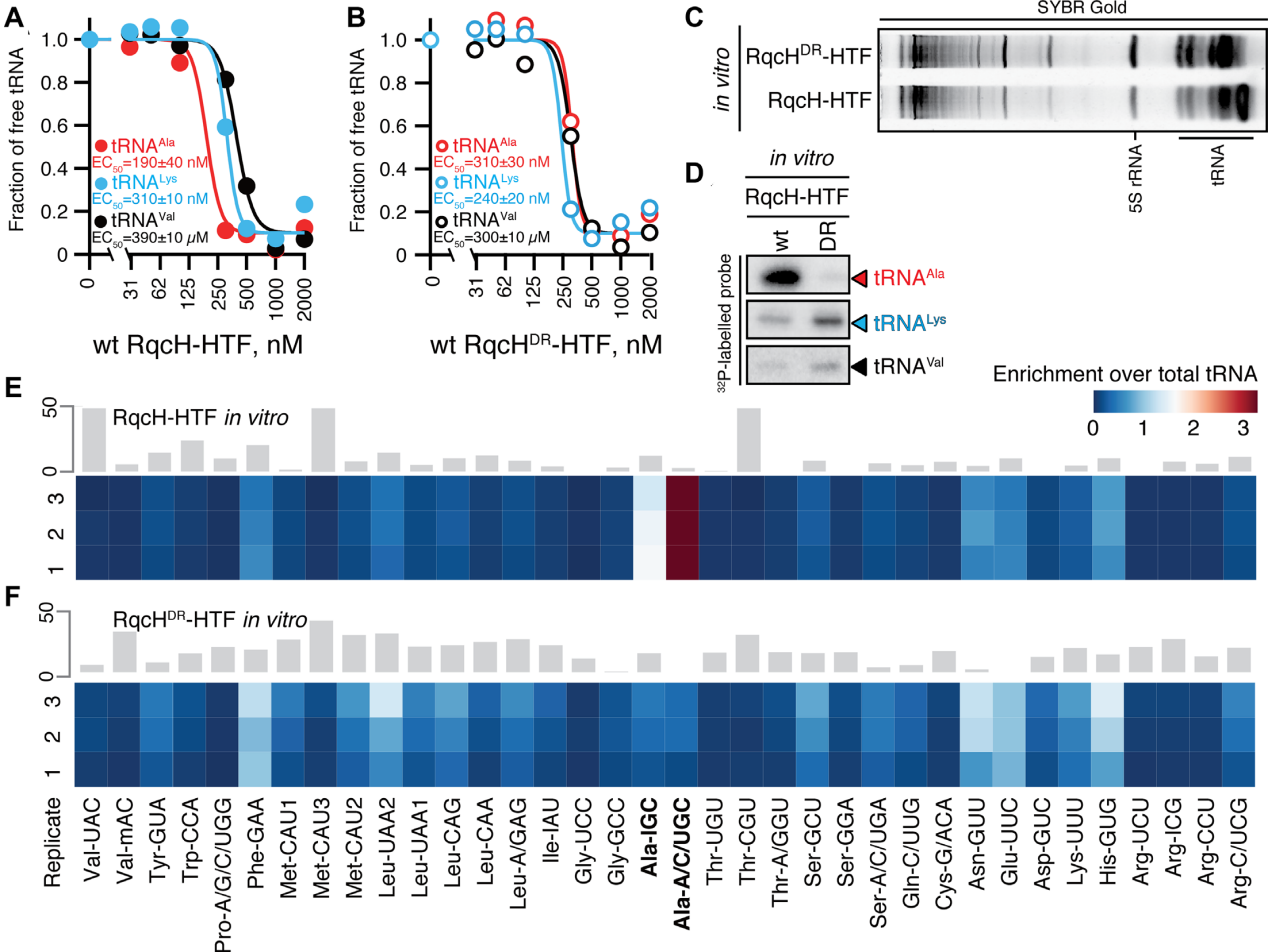
RqcH can specifically select tRNA^Ala^ from the tRNA pool off the 50S ribosome. (A, B) Complex formation between increasing concentrations of either wild-type (**A**) or DR variant (D97A R98A double substitution) (**B**) *B. subtilis* RqcH-HTF and 0.1 μM of either *B. subtilis* tRNA^Val^, *B. subtilis* tRNA^Ala^ or *E. coli* tRNA^Val^ was monitored by EMSA. 1 μM of synthetic mRNA(MVF) RNA oligonucleotide was used as a nonspecific competitor. Representative full-size EMSA gels are provided as Supplementary Figure S3. (C, D) SYBR Gold staining (**C**) and Northern blotting (**D**) analyses of RqcH-HTF:tRNA complexes isolated through co-IP of either wild-type or DR variant of RqcH-HTF preincubated with total *B. subtilis* tRNA, tBulk. For validation of northern blot probe specificity see Supplementary Figure S4. (E, F) tRNA microarray analyses of tRNAs isolated through co-IP of either wild-type (**E**) or DR variant (**F**) of RqcH-HTF preincubated with *B. subtilis* total tRNA. Grey bars represent an example of covariance analysis between replicates 1 and 3 (E) and replicates 2 and 3 (F). The Ala-IGC tRNA array probe hybridizes with tRNA^Ala(GGC)^, Ala-A/C/UGC probe hybridizes with tRNA^Ala(UGC)^ and Lys-UUU hybridizes with tRNA^Lys(UUU)^; the full reference table is provided as Supplementary Table S1. The colour key indicates the fold-enrichment of tRNAs in pulldown samples over total tRNA.

**Figure 3.**
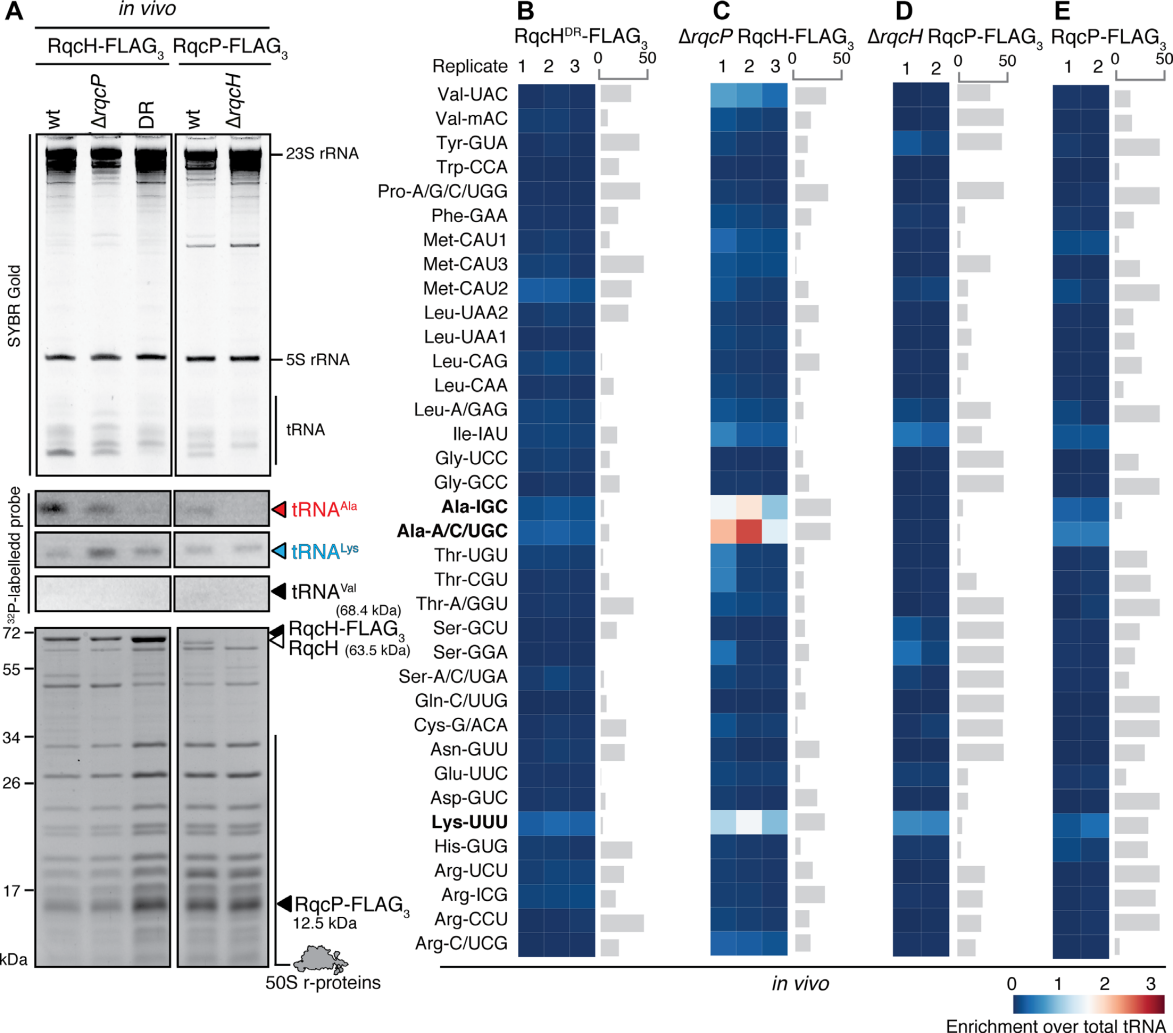
While RqcP itself does not have a specific preference for tRNA^Ala^, loss of RqcP moderately compromises tRNA^Ala^ selection by RqcH *in vivo*. (**A**) SYBR Gold staining (top), Northern blotting (middle) and SDS PAGE (bottom) analyses of 50S RQC complexes isolated through co-IP of either RqcH-FLAG_3_ or RqcP-FLAG_3_ from lysed *B. subtilis*. (B–E) tRNA microarray analyses of 50S RQC complexes isolated through co-IP of either RqcH-FLAG_3_ (**B** and **C**, three independent biological replicates) or RqcP-FLAG_3_ (**D** and **E**, two independent biological replicates) from lysed *B. subtilis*. Grey bars represent an example of covariance analysis between either replicates 1 and 3 (B), 2 and 3 (C), or 1 and 2 (D,E). The Ala-IGC tRNA array probe hybridizes with tRNA^Ala(GGC)^, Ala-A/C/UGC probe hybridizes with tRNA^Ala(UGC)^ and Lys-UUU hybridizes with tRNA^Lys(UUU)^; the full reference table is provided as Supplementary Table S1. The colour key indicates the fold-enrichment of tRNAs in pulldown samples over total tRNA.

tRNA microarrays were performed similarly to as previously described (42). A detailed protocol for the microarrays is published on protocols.io (dx.doi.org/10.17504/protocols.io.hfcb3iw). *In vivo* and *in vitro* co-immunoprecipitated tRNA samples as well as the corresponding total tRNA controls (tRNA from cell lysate for *in vivo* pulldown experiments or purified total *B. subtilis* tRNA for *in vitro* pulldown experiments, respectively) were incubated with 100 mM Tris-HCl pH = 9.0 at room temperature for 45 min, thereafter precipitated with one volume of 100 mM NaCl 100 mM NaOAc pH 4.8 as well as 2.7-volumes of absolute ethanol, resuspended in ddH_2_O and tRNAs were subjected to labeling of the invariant 3′-NCCA-ends. The IP samples were labeled with Cy3-labeled RNA/DNA while the total tRNA control samples were labeled with Atto647-labeled RNA/DNA hybrid oligonucleotide. Labeled tRNA samples were loaded as pairs (i.e. Cy3-labeled IP and Atto647-labeled total tRNA) on the same microarray containing 24 replicates of full-length tDNA probes recognizing the 36 *B. subtilis* tRNA isoacceptors (see Supplementary Table S1) and hybridized for 16 h at 60°C. The fluorescence signals of microarrays were recorded with a GenePix 4200A scanner (Molecular Devices) and statistically analyzed with in-house scripts with Python version 3.7.0. Each fluorescent signal was normalised to intrinsic standards as described (61) and presented as ratios of the IP complexes against total tRNA (i.e. Cy3 vs Atto647 signals). Thereby, while equal amounts of IP samples and total tRNAs were analyzed on each array, the IP samples originated from larger cell amounts and this was also considered by calculating the final ratios. Enrichment of tRNA species in *in vivo* and *in vitro* IP samples was calculated over corresponding total tRNA pool, i.e. either tRNA from cellular lysates or purified total *B. subtilis* tRNA, respectively. To assess the spread of the data, covariance of the signal is calculated for each single isoacceptor (represented with grey bars on the corresponding figures) which is further used to compute the confidence intervals, i.e. the spread of the data for all isoacceptors. In the case of experiments with three replicates, the confidence intervals between replicates 1 and 2, 2 and 3, 1 and 3 were 98%, 99% and 99% (for Figure 2E), 95%, 97% and 92% (Figure 2F), 96%, 96% and 97% (Figure 3B), 95%, 97% and 92% (Figure 3C) and, finally, 94%, 98% and 97% (Figure 5F). In the case of experiments with two replicates, the confidence intervals between replicates 1 and 2 were 95% (Figures 3D and 3E).

### Sucrose gradient fractionation and Western blotting

Experiments were performed as described earlier (42), with minor modifications. 50S ribosomal protein L3 was detected using anti-L3 primary antibodies provided by Fujio Kawamura (1:20,000 dilution) combined with goat anti-rabbit IgG-HRP secondary antibodies (Sigma-Aldrich, A0545; 1:10 000 dilution). ECL detection was performed using WesternBright™ Quantum (K-12042-D10, Advansta) western blotting substrate and ImageQuant LAS 4000 (GE Healthcare) imaging system.

### Poly-Ala synthesis in a reconstituted *B. subtilis* RQC system

Initiation (2 μM *B. subtilis* 50S, 3 μM RqcH-HTF, 3 μM RqcP, 2 μM f[^35^S]Met-tRNA_i_^Met^ in HEPES:Polymix buffer pH 7.0 1 mM DTT and 7.5 mM MgCl_2_) and elongation (10 μM *B. subtilis* total tRNA (tBulk), 2 μM AlaRS, 1 mM ATP, 200 μM Alanine, 1 mM DTT and 7.5 mM MgCl_2_ in HEPES:Polymix buffer pH 7.0) mixtures were separately prepared on ice. After 5 min incubation at 30°C, the two mixtures were combined and incubated at 30°C. 10 μl aliquots were taken (either after 15-min long incubation or throughout the time course), quenched with 10 μl of loading dye (7 M urea, 0.05% bromophenol blue and 100 mM NaOAc, pH 5) and resolved on acidic urea-PAGE in 1× TBE (8 M urea, 6.5% PAGE). The gel was exposed overnight, and the imaging plate was scanned on Typhoon FLA 9500 (GE Healthcare).

## RESULTS AND DISCUSSION

### RqcH specifically selects tRNA^Ala^ from the tRNA pool in the absence of the 50S subunit

Previous studies indicated that *B. subtilis* RqcH appears to specifically interact with tRNA^Ala^ and that this specificity may be mediated by the highly conserved _97_DR_98_ residues (41,42,44). To investigate this further, we used Electrophoretic Mobility Shift Assays (EMSAs) to study complex formation between *B. subtilis* RqcH – wild-type and D97A/R98A (DR) variant – and individual native deacylated tRNAs purified from *B. subtilis* bulk tRNA (Figure 2A, B and Supplementary Figure S3). We tested *B. subtilis* tRNA^Ala^ as the native RQC tRNA substrate, and, as controls, *B. subtilis* tRNA^Lys^ and *E. coli* tRNA^Val^; tRNA^Ala^ and tRNA^Val^ used for EMSA were not further purified into individual isoacceptors. While the wild-type RqcH displays a preference for tRNA^Ala^, it also binds the control tRNAs, though with lower affinity: EC_50_ tRNA^Ala^ of 190 nM versus EC_50_ tRNA^Lys^ of 310 nM and EC_50_ tRNA^Val^ of 390 nM (Figure 2A). The preference for tRNA^Ala^ is lost in the RqcH^DR^ variant, which binds all the three tRNA species with the same (low) affinity, analogous to that observed for the wild-type protein binding to non-cognate tRNA (i.e. tRNA^Val^ and tRNA^Lys^; EC_50_ 240–310 nM). The modest specificity of RqcH for tRNA^Ala^ observed in EMSAs is surprising given the pronounced selectivity for tRNA^Ala^ in *B. subtilis* RQC (41,42). The higher specificity for tRNA^Ala^*in vivo* is only evident in kinetic competition with the other tRNA species. To assess this, time-resolved studies will be necessary; while our EMSA can quantify the equilibrium affinities (*K*_D_/EC_50_), the method is not suitable for measuring the on- and off-rates of RqcH:tRNA complex formation (*k*_+1_ and *k_–_*_1_). Alternatively, it is also possible that the high selectivity is only evident when RqcH is recruited to the 50S subunit, and the preference for tRNA^Ala^ actually reflects the specificity of RqcH:50S RQC complex, rather than that of the RqcH itself.

To address the first of these possibilities, we performed a set of *in vitro* pulldown experiments using total *B. subtilis* tRNA preparation (total tRNA bulk, tBulk) and purified RqcH (C-terminally extended by the His_6_-TEV-FLAG_3_ (HTF) tag). The purified HTF-tagged wild-type RqcH or RqcH^DR^ were incubated with total tRNA, and the associated tRNA species were probed by (i) Northern blotting (Figure 2C, D; for validation of Northern blot probe specificity see Supplementary Figure S4), and (ii) tRNA microarray (Figure 2E, F). Both experiments were in agreement with each other, demonstrating strong preference of the wild-type RqcH for tRNA^Ala^. In the microarrays, we detected both tRNA^Ala(GGC)^ and tRNA^Ala(UGC)^ isoacceptors (hybridizing to the Ala-IGC and Ala-A/C/UGC probes, respectively, see Supplementary Table S1). The signal for tRNA^Ala^ is abrogated in the RqcH^DR^ variant. Notably, in the case of RqcH^DR^, the tRNA^Lys^ signal on the Northern blot (and to a lesser extent that of tRNA^Val^) is stronger than that for the wild-type protein (Figure 2D). A possible explanation is that the RQC-cognate tRNA^Ala^ efficiently out-competes tRNA^Lys^ for binding to wild-type RqcH, but not to RqcH^DR^. Finally, we validated the *in vitro* tRNA specificity results *in vivo*. Both northern blotting (Figure 3A) and tRNA microarray (Figure 3B) analyses of *in vivo* FLAG_3_ pulldown RqcH:50S complexes demonstrate that, indeed, tRNA^Ala^ specificity is lost in RqcH^DR^. Note that tRNA^Ala^ signal is clearly resolved from other RNA species on SYBR Gold gels, and the corresponding band is lacking in the RqcH^DR^ sample (Figure 3A and Supplementary Figure S5). Collectively, our results demonstrate that RqcH recruitment to the 50S subunit is not necessary for the tRNA selection by RqcH.

We also probed the role of the second RQC elongation factor, RqcP, in tRNA selection using northern blotting, SYBR Gold staining (Figure 3A and Supplementary Figure S5) and tRNA microarrays (Figure 3C–E). Compared to the *rqcP*+ sample, in the RqcH-FLAG_3_:50S pulldown from Δ*rqcP B. subtilis*, there is a moderate, but detectable decrease in abundance of both tRNA^Ala^ isoacceptors, as well as an increase of the tRNA^Lys^ signal (Figure 3C). This could be either a direct effect of intrinsic specificity of RqcP towards tRNA^Lys^, or an indirect effect of the compromised RQC elongation through RqcP depletion leading to accumulation of ‘initiation’ RQC complexes containing tRNA^Lys^. Therefore, we next probed the intrinsic tRNA selectivity of RqcP by analysing RqcP-FLAG_3_ pulldowns from both the RQC-incompetent Δ*rqcH* strain (Figure 3D) and RQC-competent *rqcH*+ strain (Figure 3E). While we see no tRNA specificity in the Δ*rqcH* pulldown samples, there is a weak signal for selection of tRNA^Lys^ of a similar magnitude and preference tRNA^Ala^ in *rqcH*+ pulldowns. This strictly-RqcH-dependent specificity signal in the *in vivo* RqcP-FLAG_3_ pulldowns can be explained by the tRNA specificity of RqcH present in RqcP-FLAG_3_-RqcH-50S (but not RqcP-FLAG_3_-RqcH-50S) RQC complexes. Our RqcP pulldown results also suggest that the decrease in tRNA^Ala^ abundance observed in the RqcH-FLAG_3_:50S pulldown from the Δ*rqcP* strain is an indirect effect of compromised RQC elongation, with the increase of the tRNA^Lys^ signal possibly reflecting the nature of the tRNA in the initial stalled 70S complexes.

### Cryo-EM structures of an *in vivo* formed RqcH^DR^-50S complex

**Figure 4.**
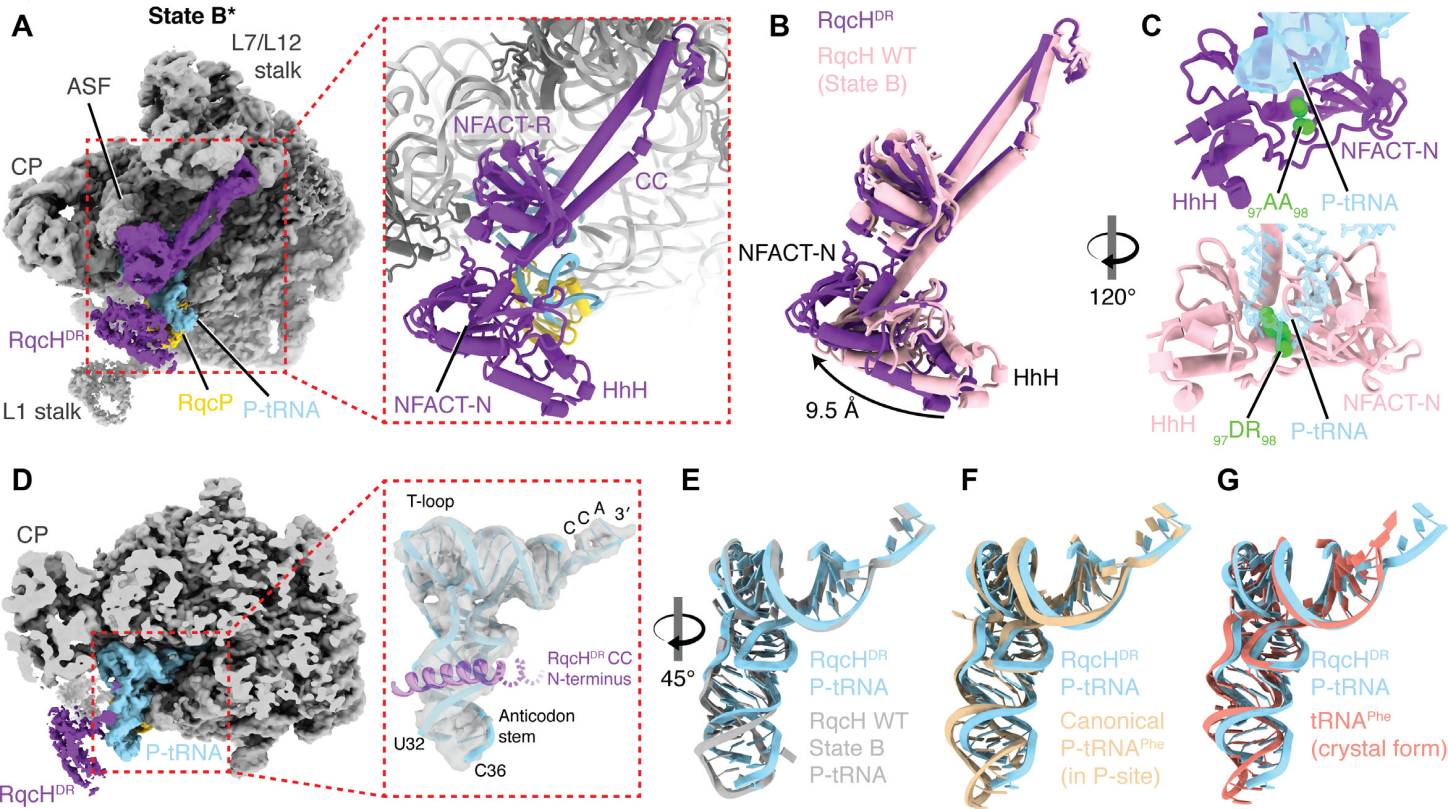
Cryo-EM structures of an *in vivo*-formed RqcH^DR^-50S complex. (**A**) View of RqcH^DR^-FLAG_3_ bound to 50S with P-tRNA and RqcP. The state broadly resembles state B described in (42). (**B**) Comparison of RqcH^DR^ (purple) with wild-type RqcH (pink) state B. (**C**) Close views, rotated compared to C, showing the position of the mutated _97_DR_98_ residues. Transparent density (top panel) or model (bottom panel) is shown for the P-tRNA anticodon stem. (**D**) View of the P-tRNA on the RqcH^DR^-bound 50S. The anticodon stem is poorly resolved, likely due to flexibility. Part of the RqcH CC N-terminus is shown. (**E**) Comparison of the P-tRNA in the RqcH^DR^ volume (light blue) with the P-tRNA in the RqcH WT-bound volume (state B, grey) (42). (**F**) As in E, except the RqcH^DR^-FLAG_3_ P-tRNA is compared to a canonical P-tRNA bound in the P-site of the ribosome (tan, PDB 6CFJ) (74). (**G**) As in E and F, except the RqcH^DR^-FLAG_3_ P-tRNA is compared to crystallised yeast tRNA^Phe^ (reddish, PDB 1EHZ) (62).

To gain the structural insight into the specific role of D97/R98 residues in tRNA^Ala^ recognition by RqcH, we determined the structure of a RqcH^DR^-50S RQC complex by cryo-EM. Following an established affinity purification procedure (42), we generated 50S RQC complexes from *B. subtilis* expressing C-terminally FLAG_3_-tagged RqcH^DR^ (D97A/R98A double substitution in NFACT-N domain) (Supplementary Figure S2). As observed previously, RqcH apparently dissociated from many particles during sample preparation, but with focussed classification we nonetheless obtained a volume with RqcH stably bound (Supplementary Figure S2, Table S3). The main state observed is broadly similar to the previously defined state B, consisting of a 50S subunit with peptidyl-tRNA in approximately the P-site, nascent chain, RqcP, and RqcH (Figure 4A). Although the overall resolution of this complex was 3.2 Å, this mostly reflected the core of the 50S subunit, as RqcH and other bound factors were less well resolved (Supplementary Figure S6). Compared to the previously observed state B (42), the RqcH^DR^ NFACT-N and HhH domains are swung away from the tRNA anticodon in the RqcH^DR^-bound structure (Figure 4B). These domains are also especially poorly resolved, indicating flexibility (Figure 4A, Supplementary Figures S6 and S7). Such flexibility is likely due to the _97_DR_98_ motif interacting with the tRNA anticodon stem in the RqcH wild-type state B structure, possibly stabilizing the major conformation (Figure 4C). Nonetheless, we were able to fit the individual domains of RqcH^DR^ into the map to create a model with sufficient confidence to examine domain-level movements. The RqcH^DR^ complex is very similar to state B* that was also observed in the wild-type RqcH reconstructions (42) (Supplementary Figure S7). The P-tRNA in the RqcH^DR^ complex had a poorly resolved anticodon (Figure 4D). Previously, wild-type RqcH has been shown to make close contact with the P-tRNA, unwinding the anticodon stem and forming interactions with the splayed anticodon (42,44). In comparison, the RqcH^DR^-bound tRNA anticodon stem was not as distorted (Figure 4E). However, compared to a canonical P-tRNA or free crystallised tRNA (62), the RqcH^DR^-bound P-tRNA was still somewhat distorted, perhaps as a result of other regions of RqcH, such as a N-terminus of the coiled-coil domain, that contact the tRNA (Figure 4F, G) (42,44). Collectively, the structure suggests that the mutation of _97_DR_98_ motif to _97_AA_98_ destabilizes the interaction of the NFACT-N domain of RqcH^DR^ with the anticodon stem loop of the P-tRNA, thereby supporting the hypothesis that this interaction is critical for the specificity of RqcH for tRNA^Ala^. In the available structures so far, RqcH _97_DR_98_ has been observed either to interact with G35 at the central position of the tRNA anticodon, which is common to several *B. subtilis* tRNAs, or not interact with the tRNA at all. It is therefore unlikely that these conformations alone can account for the tRNA specificity of RqcH. RqcH and tRNA^Ala^ may form a complex with an alternative conformation when not bound in complex with the 50S subunit.

### Ribosomal protein uL11 is important for RQC functionality

**Figure 5.**
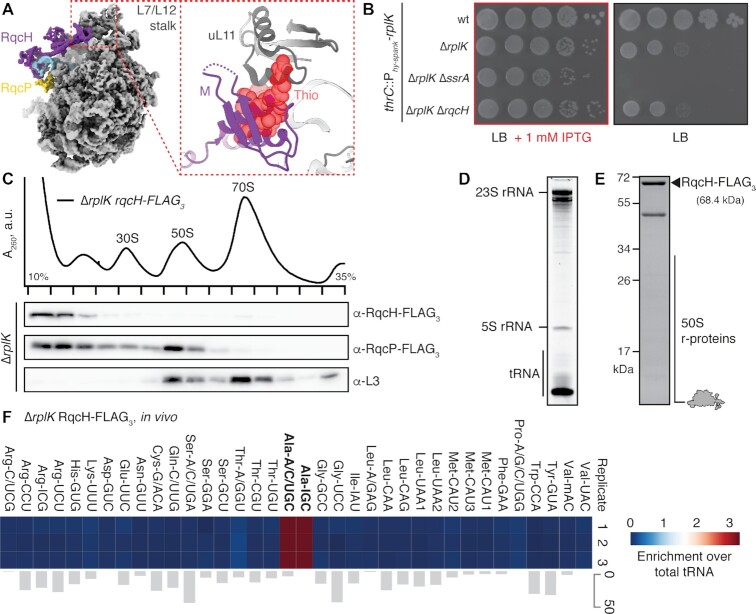
Ribosomal protein uL11 is important for RqcH recruitment to 50S and for RQC functionality. (**A**) Overview of wild-type RqcH (state B, PDB 7AS8, EMD-11889) bound to the 50S-P-tRNA complex with focus on the L7/L12 stalk base. Right, close view of the RqcH M hairpin interacting with uL11. Antibiotic thiostrepton (Thio, in red) is modeled based on PDB 3CF5 (63). (**B**) *B. subtilis* strains expressing ribosomal uL11 (*rplK*) under the control of IPTG-inducible P*_hy__−__spank_* promoter in wild-type, Δ*rplK*, Δ*rplK* Δ*ssrA* and Δ*rplK* Δ*rqcH* backgrounds were grown in either uL11-inducing (LB supplemented with 1 mM IPTG, left) or non-inducing (LB, right) conditions. Plates were scored after 18 hours at 37°C. (**C**) Sucrose gradient sedimentation and anti-FLAG_3_ immunoblotting of either RqcH-FLAG_3_ or RqcP-FLAG_3_ expressed in Δ*rplK* background. (D–F) SYBR Gold staining (**D**), SDS PAGE (**E**) and tRNA microarray analyses (**F**) of 50S RQC complexes isolated through co-IP of RqcH-FLAG_3_ from lysed Δ*rplK B. subtilis*. Three independent biological replicates of tRNA microarray analyses are shown. Grey bars represent an example of covariance analysis between replicates 1 and 3. The Ala-IGC tRNA array probe hybridizes with tRNA^Ala(GGC)^, Ala-A/C/UGC probe hybridizes with tRNA^Ala(UGC)^ and Lys-UUU hybridizes with tRNA^Lys(UUU)^; the full reference table is provided as Supplementary Table S1. The colour key indicates the fold-enrichment of tRNAs in pulldown samples over the total tRNA.

In the cryo-EM structure of the RqcH-50S RQC complex, we observed strong interaction between the M domain of RqcH and the uL11 stalk base (Figure 5A), as observed previously for the wild-type RqcH 50S complexes (42,44). This interaction appeared to be critical for RqcH function since treatment with the antibiotic thiostrepton that interacts with uL11 (63), and has an overlapping binding site with RqcH M domain (inset to Figure 5A), destabilised interaction of RqcH with the 50S RQC complex (42). To directly investigate the importance of uL11 for RQC function, we constructed a *B. subtilis* strain in which uL11 is expressed under the control of an IPTG-inducible P*_hy__−__spank_* promoter (64), while the genomic copy of *rplK* encoding uL11 is disrupted (Δ*rplK*). This allowed conditional depletion of uL11 in bacteria that are grown in the absence of an inducer. uL11 depletion results in a pronounced growth defect as seen in Figure 5B. When we combined the depletion of uL11 with *rqcH* disruption, we did not observe a further synthetic growth defect. This lack of genetic interaction is consistent with RQC functionality being already lost in the uL11-depleted strain. By contrast, simultaneous loss in functionality of both RQC and trans-translation ribosome rescue systems renders *B. subtilis* sensitive to elevated temperature and antibiotics targeting protein synthesis, such as tetracycline (41,42,44). This synergetic growth defect is commonly used for probing RQC functionality in an Δ*ssrA* background (41,42,44). Indeed, we observed a strong genetic interaction of *rplK* with *ssrA*: uL11 depletion in Δ*ssrA* Δ*rplK* genetic background results in the synthetic lethality of the strain (Figure 5B). This is likely due to the proteotoxic stress due to a defunct RQC and trans-translation, combined with the general translation defect due to loss of uL11.

Next, we assessed the effect of uL11 on the recruitment of RQC factors to the 50S RQC complex using sucrose gradient centrifugation and Western blotting against the FLAG-tagged RqcH or RqcP factors (Figure 5C). While RqcP remains associated with ΔuL11 50S, RqcH is lost from the 50S fractions in the Δ*rplK* strain (Figure 5C), strongly suggesting that uL11 plays a key role in RqcH recruitment to 50S, and, therefore, RQC function. In good agreement with decreased affinity of RqcH to 50S lacking uL11, characterization of RqcH-FLAG_3_ pulldown samples revealed relatively low abundance of both rRNA and r-proteins in comparison to pulldowns from wild-type strain (Figure 5D, E, a side-by-side comparison is shown on Supplementary Figure S5). By contrast, the strong band consistent with tRNA remained (Figure 5D), in agreement with RqcH being able to specifically recruit tRNA^Ala^ in the absence of the 50S subunit. tRNA microarray analysis of the RqcH-FLAG_3_ pulldown samples from *B. subtilis* Δ*rplK* revealed that indeed RqcH specifically interacts with both tRNA^Ala^ isoacceptors (Figure 5F), as seen previously for tRNA microarray analysis of RqcH 50S complexes (42).

### Polypeptide release dissociates RqcP from RqcP-RqcH-50S RQC complexes

**Figure 6.**
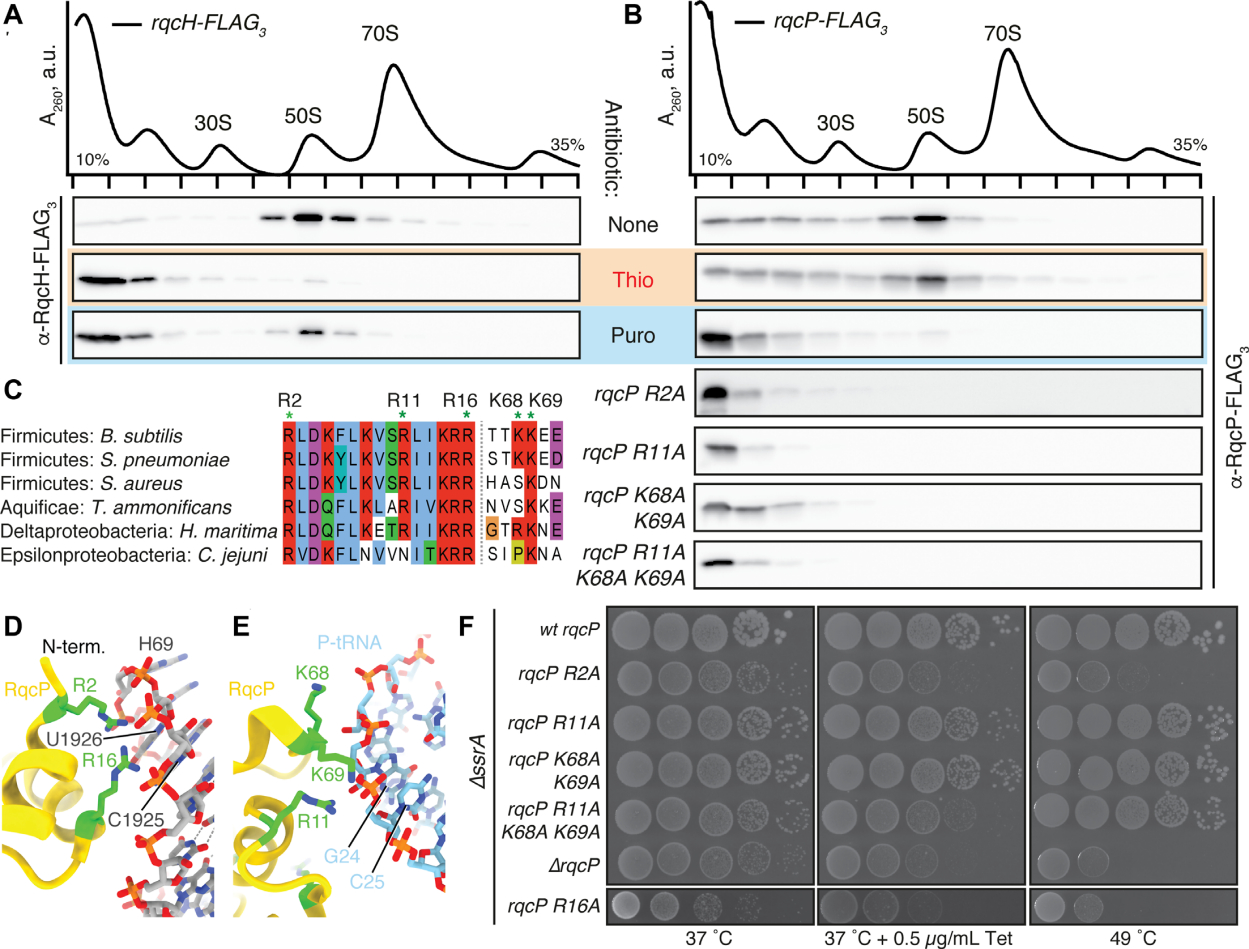
Interaction of RqcP with 23S rRNA H69, but not with P-site tRNA^Ala^, is essential for RQC functionality. (A, B) Sucrose gradient sedimentation and anti-FLAG_3_ immunoblotting analysis of *B. subtilis* strains expressing either wild-type RqcH-FLAG_3_ (**A**) or RqcP-FLAG_3_, wild-type and substituted variants (**B**). As indicated on the figure, the antibiotics were added after cell lysis: thiostrepton (Thio, 50 μM) and puromycin (Puro, 1 mM). (**C**) Sequence alignment of RqcP/Hsp15 homologs from diverse bacteria. (D, E) View of RqcP from state B interacting with (**D**) 23S rRNA H69 or (**E**) the P-tRNA (PDB 7AS8). Residues selected for mutation are coloured green. 23S rRNA nucleotides are numbered according to *E. coli* numbering. (**F**) Synthetic growth defects caused by amino acid substitutions in *rqcP*, or *rqcP* deletion in a Δ*ssrA* background. 10-fold serial dilutions were spotted onto LB agar plates and incubated for 18 hours at 37°C (left), 49°C (right) or 37°C in the presence 0.5 μg/ml tetracycline (Tet, middle).

Our finding that uL11 is critical for RqcH, but not RqcP, interaction with the 50S subunit (Figure 5C) suggested that binding of RqcP to the 50S subunit is independent of RqcH. This is consistent with cryo-EM analysis where P-tRNA-50S complexes containing RqcP but lacking RqcH are observed (Supplementary Figure S2) (42). To pursue this further, we employed sucrose density gradient centrifugation and Western blotting to monitor the association of both RqcH and RqcP in the presence of the antibiotics thiostrepton and puromycin (Figure 6A,B). As before (42), we observed that thiostrepton abolished the interaction of RqcH with the 50S subunit (Figure 6A), whereas here we show that RqcP remains unaffected (Figure 6B). By contrast, the addition of puromycin, which mediates release of the polypeptide chain from the P-tRNA (65), led to a modest reduction in RqcH binding (Figure 6A), as observed previously (42), but more strikingly, resulted in a dramatic reduction of the RqcP band within the 50S fraction (Figure 6B). Since release of the nascent polypeptide chain by puromycin is likely to have a destabilizing effect on the binding of P-tRNA to the 50S subunit, these findings suggest that binding of RqcP, and to a lesser extent RqcH, is stabilized by the presence of the peptidyl-tRNA in the P-site.

### Stable interaction of RqcP with 23S rRNA H69, but not with P-site tRNA^Ala^ is essential for RQC functionality

In the 50S RQC complex, RqcP forms a network of contacts with P-site tRNA, but also the 23S rRNA helix 69 (H69) (42,44). Interactions with H69 are mediated by conserved Arg2 and Arg16 of RqcP, whereas contacts with P-tRNA are formed by Arg11, Lys68 and Lys69 (Figure 6C–E). To date, only Arg16 of RqcP was assessed experimentally for its functional role, and the residue was shown to be crucial because Arg16Ala substitution abolished RqcP association with the 50S subunit and, when introduced in the Δ*ssrA* background, *rqcP R16A* phenocopies Δ*ssrA* Δ*rqcP* double deletion (42).

We first probed the importance of Arg2, Arg11, Lys68 and Lys69 in RqcP association with the 50S through sucrose gradient sedimentation experiments. As seen in Figure 6B, the association with 50S was compromised for all of the tested RqcP variants, suggesting that the contacts of RqcP with both H69 and the P-site tRNA are both important for establishing a stable interaction with the large subunit. Next, we assessed the functional importance of Arg2, Arg11, Lys68 and Lys69 *in vivo* by following the growth of Δ*ssrA B. subtilis* strains expressing substituted RqcP variants. The cells were grown either in normal conditions (37°C), at elevated temperature (49°C), or in the presence of low concentrations of tetracycline (Figure 6F). Similar to R16A (42), the R2A substitution phenocopies the loss of RqcP (Δ*rqcP*), thereby re-emphasizing the importance of the interaction of RqcP with H69 of the 23S rRNA for RQC functionality. By contrast, there appears to be redundancy in the interactions between RqcP and the P-site tRNA: Δ*ssrA B. subtilis* strains expressing single R11A-substituted and the double K68A/K69A-substituted RqcP variants display wild-type-like phenotypes, only with the triple R11A/K68A/K69A substitution resulting in a growth defect (Figure 6F). The apparent discrepancy between the loss of interaction of the R11A and K68A/K69A variants with the 50S subunit (Figure 6B) and the lack of effect in the growth assays (Figure 6F) is likely due to the non-equilibrium nature of sucrose gradient sedimentation experiments, i.e. the lack of association does not indicate an absence of the interaction in the cell, but rather means that the complex is not retained during the 3-hour-long centrifugation.

### RqcP is a processivity factor essential for poly-alanine synthesis during RQC elongation

**Figure 7.**
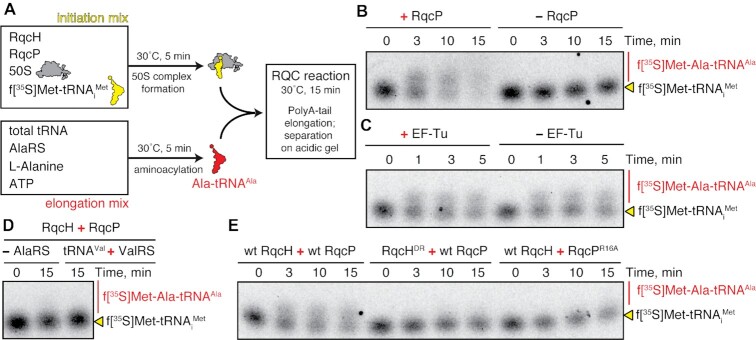
RqcP is a processivity factor essential for poly-Ala synthesis in a biochemically reconstituted RQC system. (**A**) Schematics of the experimental setup. Separate initiation and elongation mixtures were prepared in HEPES:Polymix buffer (7.5 Mg^2+^ pH 7.0) and incubated at 30°C for 5 min before combining. After an additional 15 minute-long incubation, tRNA species were resolved in a bis-tris gel and visualised by phosphoimaging. (**B**) Formation of Ala-tRNA^Ala^ in the reconstituted RQC system is abrogated upon omission of RqcP. (**C**) Addition of canonical translation elongation factor EF-Tu and 1 mM GTP does not affect the efficiency of Ala-tRNA^Ala^ synthesis. (**D**) No poly-Ala synthesis is observed either upon omission of AlaRS or in the presence of tRNA^Val^, Valyl-tRNA synthetase (ValRS) and l-valine. (**E**) Neither RqcH^DR^ nor RqcP^R16A^ support Ala-tRNA^Ala^ formation in the reconstituted RQC system.

Previous studies have shown using *in vivo* approaches that RqcP phenocopies RqcH in the Δ*ssrA* background (42,44) and that deletion of *rqcP* leads to a loss of poly-alanine tailing of a non-stop reporter construct (44), collectively indicating that RqcP has a critical role in bacterial RQC. However, it still remains unclear whether RqcH and RqcP are necessary and sufficient to mediate polyalanine tailing of 50S–peptidyl-tRNA complexes. To investigate this, we undertook to establish an *in vitro* polyalanine tailing system using only purified RQC factors, as has been previously performed for canonical translation (66,67). To initiate RQC, we loaded 50S subunits with radioactively labeled formylated initiator tRNA, ^35^S-fMet-tRNA_i_^Met^, which has preferential affinity to the ribosomal P-site (68). We hypothesized that the fMet–tRNA–50S complex would mimic the peptidyl-tRNA 50S complexes that arise during RQC and thereby act as a substrate for RqcH and RqcP (Figure 7A). In parallel, we assembled a separate reaction containing Ala-tRNA^Ala^ from *B. subtilis* total tRNA that had been incubated with *B. subtilis* alanyl-tRNA synthetase (AlaRS), ATP and l-alanine at 30°C. Since the Mg^2+^ concentration is a major determinant of tRNA affinity to the ribosome (69,70), we increased the concentration to 7.5 mM in our HEPES:Polymix-based buffer system (47) to find the optimal balance between tRNA affinity (for efficient Ala-tRNA^Ala^ recruitment to 50S RQC complex) and dynamics (for efficient RQC elongation), and adjusted the pH to 7.0 in order to stabilise aminoacyl-tRNA. After preincubation the two mixtures were combined, the resultant reaction was further incubated for 30 minutes at 30°C, tRNA species were resolved in a bis-tris gel and then visualised by phosphoimaging.

In the reaction lacking RqcP, we only detect ^35^S-labeled fMet-tRNA_i_^Met^, whereas in the presence of RqcP there is an up-shift in the ^35^S-labeled fMet signal, which is consistent with the polyalanine tailing of the fMet (Figure 7B), thus directly demonstrating the role of RqcP as an essential processivity factor for RQC elongation. No effect of the efficiency of poly-Ala synthesis was observed in the RQC reaction that was additionally supplemented with canonical translation elongation factor EF-Tu and 1 mM GTP (Figure 7C), suggesting that EF-Tu does not play a role in delivery of Ala-tRNA to the 50S subunit and that tailing proceeds independently of GTP. Moreover, no poly-alanine tailing was observed when the aminoacylation mix was replaced with the presence of tRNA^Val^, valyl-tRNA synthetase (ValRS), ATP and l-valine (Figure 7D), which is consistent with the strong preference of RqcH for tRNA^Ala^. Similarly, no activity was detected when AlaRS was omitted (Figure 7D). Finally, our reconstituted RQC system faithfully reproduces the effects of loss-of-function substitutions in RqcH (RqcH^DR^ variant compromised in tRNA^Ala^ selection) and RqcP (RqcP^R16A^ variant compromised in interaction with the H69 rRNA element), since no poly-alanine tailing was observed when replacing the wild-type RqcH and RqcP with these variants (Figure 7E). Collectively, our biochemical results establish that the combination of RQC factors RqcP and RqcH is both necessary and sufficient to drive polyalanine synthesis on isolated 50S subunits in the absence of canonical translation elongation factors.

## CONCLUSIONS AND PERSPECTIVE

This study supports the proposed molecular mechanism of C-terminal alanine tailing mediated by the concerted action of RqcH and RqcP (Figure 1D) (42). Importantly, it also opens up several research directions for follow-up investigations. What is the significance of tRNA^Lys^ enrichment in 50S RQC complexes compromised in RQC elongation due to the DR substitution in the NFACT-N domain of RqcH or loss of RqcP? It is tempting to speculate that tRNA^Lys^ is enriched in these complexes due to ribosomal stalling on lysine residues triggering RQC. Indeed, a recent analysis of ribosome profiling data suggests that the presence of P-site Lys residues encoded by AAA codons are associated with a moderate slowing down of translation elongation in *E. coli* (71). However, no Lys-associated ribosomal stalling signal was detected in a *B. subtilis* study using 5Pseq (72). Therefore, while tempting, this hypothesis requires further investigation. Second is the question of a potential C-terminal-tailing processivity factor in eukaryotic RQC. Here we directly demonstrate *in vitro* that S4 homologue Hsp15-family protein RqcP is, indeed, essential to drive the poly-alanine tailing by 50S-associated RqcH/Rqc2/NEMF in a minimal bacterial system. This indicates that an analogous yet-to-be-discovered factor could be also cooperating with Rqc2/NEMF in archaea and eukaryotes. Additionally, the establishment of a reconstituted bacterial RQC system provides researchers with a powerful tool for activity-driven discovery of additional bacterial RQC factors, either by directly testing candidate factors identified through structural and genetic studies or through fractionation of cellular lysates. Currently, it is unclear which cellular factors split the stalled 70S ribosomes to generate the 50S RQC substrate and which factors terminate the RQC-mediated poly-alanine addition to release the tagged polypeptide. Our genetic experiments suggest that ribosome-splitting factor HflX (73) is not essential for RQC in live cells since we detect no synthetic growth defect upon the simultaneous deletion of *hflX* and *ssrA* (Supplementary Figure S8). A fully reconstituted biochemical RQC system could be instrumental for deconvoluting the redundancies and overlapping functions of the factors involved. Finally, an analogous eukaryotic RQC system could be established using 60S ribosomal subunits, Rqc2/NEMF and aminoacylated tRNAs to enable activity-driven discovery of additional RQC factors.

## DATA AVAILABILITY

The cryo-EM map of the RqcH^DR^-50S complex and the associated molecular model have been deposited in the Protein Data Bank and Electron Microscopy Data Bank with the accession codes EMPIAR-10726, EMD-13017 and PDB-7OPE, respectively. The tRNA microarray data have been deposited in Gene Expression Omnibus (GEO) database under the accession number GSE174254.

## Supplementary Material

gkab589_Supplemental_Files
