# Supplementary material for: RqcH and RqcP catalyze processive poly-alanine synthesis in a reconstituted ribosome-associated quality control system
Source: Nucleic Acids Res. 2021 Jul 13;49(14):8355–69. doi: 10.1093/nar/gkab589 (PMC8373112; doi:10.1093/nar/gkab589)
Supplement: gkab589_Supplemental_Files [file gkab589_supplemental_files.zip › Takada_SI.pdf]

SUPPLEMENTARY ONLINE MATERIALS

for

RqcH and RqcP catalyze processive poly-alanine synthesis in a reconstituted ribosome-associated quality control system

Hiraku Takada[1,2,3,*], Caillan Crowe-McAuliffe[4], Christine Polte[4], Zhanna Yu. Sidorova[5,6], Victoriia Murina[2,3], Gemma C. Atkinson[8], Andrey L. Konevega[5,7,8], Zoya Ignatova[4], Daniel N. Wilson[4,*], Vasili Hauryliuk[2,3,9,10,*]

[1] Faculty of Life Sciences, Kyoto Sangyo University, Kamigamo, Motoyama, Kita-ku, Kyoto 603-8555, Japan

[2] Department of Molecular Biology, Umeå University, 90187 Umeå, Sweden

[3] Laboratory for Molecular Infection Medicine Sweden (MIMS), Umeå University, 90187 Umeå, Sweden

[4] Institute for Biochemistry and Molecular Biology, University of Hamburg, 20146 Hamburg, Germany

[5] Petersburg Nuclear Physics Institute named by B.P. Konstantinov of National Research Centre "Kurchatov Institute", 188300 Gatchina, Russia

[6] Russian Research Institute of Hematology and Transfusiology of FMBA, 191024 Saint Petersburg, Russia

[7] Peter the Great St. Petersburg Polytechnic University, 195251 Saint Petersburg, Russia

[8] National Research Centre "Kurchatov Institute", 123182 Moscow, Russia

[9] Department of Experimental Medical Science, Lund University, 221 00 Lund, Sweden

[10] University of Tartu, Institute of Technology, 50411 Tartu, Estonia

Supplementary methods

Preparation of B. subtilis *and* E. coli *tRNAs*

B. subtilis *total tRNA*. Total transfer RNA from wild-type 168 *B. subtilis* was isolated as follows. *B. subtilis* was grown in 2xLB to early stationary phase, 70 g of cells were collected by centrifugation, resuspended in 200 mL H_2O, combined with an equal volume of phenol and stirred for 1 hour. After centrifugation at 4000 g for 30 minutes the aqueous phase was taken, and the organic phase was re-extracted twice. The aqueous phase combined and mixed with 1/10 of volume 20% potassium acetate, and nucleic acids were precipitated with 2 volumes of ethanol. The pellet was resuspended in 100 mL of water, and after the NaCl concentration was brought to 1 M, the mixture was stirred for 1 hour at 4 °C. The debris was removed by centrifugation (1 hour at 10,000-15,000 g) and discarded, while the supernatant was taken and precipitated with ethanol. The resultant pellet was resuspended in 100 mL of 0.3 M sodium acetate pH 7.0, and re-extracted with 0.4 volume isopropanol. The supernatant was mixed with 0.6 volumes cold isopropanol, the RNA collected by centrifugation (1 hour at 4,000 g) and the RNA pellet was resuspended in 100 mL of buffer A (0.2 M NaCl, 50 mM NaAc, 10 mM $MgCl_2$, pH 4.5-5.0). The resultant solution was loaded onto a Q-Toyopearl column pre-equilibrated in the same buffer. After the column was washed with 2 volumes of buffer A, the tRNA was eluted with buffer B (1.0 M NaCl, 50 mM NaAc, 10 mM $MgCl_2$, pH 4.5-5.0), the pH adjusted to pH 8.5, tRNA was deacylated by incubation at 37 °C for 40 minutes at and precipitated by ethanol. The pellet was dissolved in 10 mL of ddH_2O and the tRNA quality as assessed by analytical by HPLC chromatography on Lichrospher RP18 250-4 column in 5-15% gradient of ethanol in buffer A.

B. subtilis *$tRNA^{Ala}$ and $tRNA^{Lys}$*. Individual tRNA species were prepared as per (1) with minor modifications. Briefly, total *B. subtilis* tRNA was aminoacylated with for 30 minutes at 37 °C using either recombinant *B. subtilis* Ala-RS or *E. coli* Lys-RS (30 µg of aa-RS per 1 mL of reaction mixture; 50 mM Tris HCl pH 7.5, 70 mM NH_4Cl, 30 mM KCl, 7 mM $MgCl_2$, 2 mM DTT, 3mM ATP, 0.5 mM of amino acid). Elongation factor Tu (EF-Tu) was preincubated for 15 minutes with 1 mM GTP, 3 mM PEP, 10 µg/mL pyruvate kinase in buffer C (50 mM Tris HCl pH 7.5, 70 mM NH_4Cl, 30 mM KCl, 7 mM $MgCl_2$) to convert EF-Tu from GDP- to GTP-bound form. Aminoacylated tRNA was supplemented with two-fold excess of EF-Tu-GTP to form a ternary complex (aa-tRNA–EF-Tu–GTP) for 5 min at 37 °C, and then placed on ice. The ternary complex was loaded on 1 mL column Protino@NI-IDA (MAcherey Nagel), the column washed with 3 volumes of buffer C (50 mM Tris HCl pH 7.5, 70 mM NH_4Cl, 30 mM KCl, 7 mM $MgCl_2$), and the ternary complex was eluted with 3 volumes of buffer D (25 mM Tris HCl pH 7.5, 50 mM KCl, 10 mM $MgCl_2$, 2.5% glycerol, 300 mM imidazole). The eluted ternary complex was dissociated by addition of potassium acetate (pH 5.0) to final concentration of 0.2 M, aminoacylated

tRNA was phenol-extracted and precipitated with ethanol. The tRNA pellet was dissolved in ddH_2O, deacylated as described above, re-precipitated, re-dissolved in 0.1 mL of ddH_2O and stored at –80 °C.

E. coli *f[^{35}S]Met-tRNA$_i^{Met}$*. f[^{35}S]Met-tRNA$_i^{Met}$ was produced from *E. coli* tRNA$_i^{Met}$ as described previously (2).

Protein expression and purification

B. subtilis *alanyl-tRNA synthetase*, *N-terminally His$_6$-tagged AlaS* (VHp772, pET24d-His$_6$-AlaRS). The protein was overexpressed in freshly transformed *E. coli* BL21 DE3 Rosetta (Novagen). Fresh transformants were inoculated to a final OD_{600} of 0.05 in the LB medium (2000 mL) supplemented with 100 µg/mL kanamycin. The cultures were grown at 37 °C until an OD_{600} of 0.5, induced with 1 mM IPTG (final concentration) and grown for additional 1.5 hours at 30 °C. The cells were harvested by centrifugation and resuspended in binding buffer (1000 mM NH_4Cl, 10 mM MgC_{l2}, 10 mM imidazole, 10% glycerol, 8 mM β-mercaptoethanol, 25 mM HEPES:KOH pH 7.5) supplemented with 0.1 mM PMSF and 1 U/mL of DNase I. Cells were lysed by one passage through a high-pressure cell disrupter (Stansted Fluid Power, 150 MPa), cell debris was removed by centrifugation (25,000 rpm for 40 min, JA-25.50 Beckman Coulter rotor) and clarified lysate was taken for protein purification. Clarified cell lysate was filtered through a 0.2 µm syringe filter and loaded onto a 5 mL HisTrap HP column pre-equilibrated in binding buffer. The column was washed with 5 CV of binding buffer, and the protein was eluted with a linear gradient (6 CV, 0-100% elution buffer) of elution buffer (1000 mM NH_4Cl, 10 mM MgC_{l2}, 500 mM imidazole, 10% glycerol, 8 mM β-mercaptoethanol, 25 mM HEPES:KOH pH 7.5). Fractions most enriched in His$_6$-AlaS (≈30-45% elution buffer) were pooled, totalling approximately 8 mL. The sample was diluted 4 times with dilution buffer (5 mM $MgCl_2$, 10% glycerol, 1 mM β-mercaptoethanol, 25 mM Tris-HCl pH 7.5) and loaded on HiPrep Q XL 16/10 column pre-equilibrated with binding buffer (150 mM NaCl, 5 mM $MgCl_2$, 10% glycerol, 1 mM β-mercaptoethanol, 25 mM Tris-HCl pH 7.5) and the protein was eluted with a linear gradient (3 CV, 0-100% elution buffer) of elution buffer (1000 mM NaCl, 5 mM $MgCl_2$, 10% glycerol, 1 mM β-mercaptoethanol, 25 mM Tris-HCl pH 7.5). Fractions most enriched in His$_6$-AlaS (≈65-75% elution buffer) were pooled, totalling approximately 8 mL. The fractions containing His$_6$-AlaS were pooled and applied on HiPrep 10/26 desalting column (GE Healthcare) pre-equilibrated with storage buffer (300 mM KCl, 15 mM $MgCl_2$, 10% glycerol, 1 mM β-mercaptoethanol, 25 mM HEPES:KOH pH 7.5). The fractions containing His$_6$-AlaS were collected and concentrated in an Amicon Ultra (Millipore) centrifugal filter device (cut-off 50 kDa). Protein preparations were aliquoted, frozen in liquid nitrogen.

Saccharomyces cerevisiae valyl-tRNA synthetase, His$_6$-tagged. The protein was overexpressed and purified as described earlier (3), the aminoacylation activity towards *E. coli* tRNA^{Val} was demonstrated in the original report.

B. subtilis *EF-Tu, C-terminally His$_6$-tagged* (VHp733, pET24d-tufA-TEV-His$_6$). The protein was overexpressed in freshly transformed *E. coli* BL21 DE3 Rosetta (Novagen). Fresh transformants were inoculated to a final OD_{600} of 0.05 in the LB medium (800 mL) supplemented with 100 µg/mL kanamycin. The cultures were grown at 37 °C until an OD_{600} of 0.5, induced with 1 mM IPTG (final concentration) and grown for additional 1.5 hours at 30 °C. The cells were harvested by centrifugation and resuspended in binding buffer (500 mM NaCl, 5 mM $MgCl_2$, 10 mM imidazole, 10% glycerol, 25 µM GDP, 1 mM β-mercaptoethanol, 25 mM Tris-HCl pH 7.5) supplemented with 0.1 mM PMSF and 1 U/mL of DNase I. Cells were lysed by one passage through a high-pressure cell disrupter (Stansted Fluid Power, 150 MPa), cell debris was removed by centrifugation (25,000 rpm for 40 min, JA-25.50 Beckman Coulter rotor) and clarified lysate was taken for protein purification. Clarified cell lysate was filtered through a 0.2 µm syringe filter and loaded onto a 5 mL HisTrap HP column pre-equilibrated in binding buffer. The column was washed with 5 CV of wash buffer (1000 mM NaCl, 5 mM $MgCl_2$, 10 mM imidazole, 10% glycerol, 25 µM GDP, 1 mM β-mercaptoethanol, 25 mM Tris-HCl pH 7.5), and the protein was eluted with a linear gradient (6 CV, 0-100% elution buffer) of elution buffer (500 mM NaCl, 5 mM $MgCl_2$,500 mM imidazole, 10% glycerol, 25 µM GDP, 1 mM β-mercaptoethanol, 25 mM Tris-HCl pH 7.5). Fractions most enriched in EF-Tu-His$_6$ (≈35-65% elution buffer) were pooled, totalling approximately 8 mL. The sample was diluted 4 times with dilution buffer (5 mM $MgCl_2$, 10% glycerol, 25 µM GDP, 1 mM β-mercaptoethanol, 25 mM Tris-HCl pH 7.5) and loaded on HiPrep Q XL 16/10 column pre-equilibrated with binding buffer (150 mM NaCl, 5 mM $MgCl_2$, 10% glycerol, 25 µM GDP, 1 mM β-mercaptoethanol, 25 mM Tris-HCl pH 7.5) and the protein was eluted with a linear gradient (3 CV, 0-100% elution buffer) of elution buffer (1000 mM NaCl, 5 mM $MgCl_2$, 10% glycerol, 25 µM GDP, 1 mM β-mercaptoethanol, 25 mM Tris-HCl pH 7.5). Fractions most enriched in EF-Tu-His$_6$ (≈55-75% elution buffer) were pooled, totalling approximately 12 mL. The fractions containing EF-Tu-His$_6$ were pooled and applied on HiPrep 10/26 desalting column (GE Healthcare) pre-equilibrated with storage buffer (400 mM KCl, 5 mM $MgCl_2$, 10% glycerol, 25 µM GDP, 1 mM β-mercaptoethanol, 50 mM HEPES:KOH pH 8). The fractions containing EF-Tu-His$_6$ were collected and concentrated in an Amicon Ultra (Millipore) centrifugal filter device (cut-off 30 kDa). Protein preparations were aliquoted, frozen in liquid nitrogen.

B. subtilis *RqcH, C-terminally HTF-tagged, wild-type* (VHp982, pET24d-rqcH-HTF) *and DR variant (D97A R98A)* (VHp983, pET24d-rqcHDR-HTF). Both proteins were overexpressed in freshly transformed *E. coli*

BL21 DE3 Rosetta (Novagen). Fresh transformants were inoculated to a final OD_{600} of 0.05 in the LB medium (800 mL) supplemented with 100 µg/mL kanamycin. The cultures were grown at 37 °C until an OD_{600} of 0.5, induced with 1 mM IPTG (final concentration) and grown for additional 1.5 hours at 30 °C. The cells were harvested by centrifugation and resuspended in binding buffer (400 mM NaCl, 5 mM MgC_{l2}, 20 mM imidazole, 10% glycerol, 4 mM β-mercaptoethanol, 25 mM HEPES:KOH pH 8) supplemented with 0.1 mM PMSF and 1 U/mL of DNase I. Cells were lysed by one passage through a high-pressure cell disrupter (Stansted Fluid Power, 150 MPa), cell debris was removed by centrifugation (25,000 rpm for 40 min, JA-25.50 Beckman Coulter rotor) and clarified lysate was taken for protein purification. Clarified cell lysate was filtered through a 0.2 µm syringe filter and loaded onto a 5 mL HisTrap HP column pre-equilibrated in binding buffer. The column was washed with 5 CV of wash buffer (2000 mM NaCl, 5 mM $MgCl_2$, 20 mM imidazole, 10% glycerol, 4 mM β-mercaptoethanol, 25 mM HEPES:KOH pH 8), and the protein was eluted with a linear gradient (6 CV, 0-100% elution buffer) of elution buffer (400 mM NaCl, 5 mM $MgCl_2$,500 mM imidazole, 10% glycerol, 4 mM β-mercaptoethanol, 25 mM HEPES:KOH pH8). Fractions most enriched in RqcH-HTF (≈30-50% elution buffer) were pooled, totalling approximately 5 mL. The sample was loaded on a HiLoad 16/600 Superdex 200 pg column pre-equilibrated with Gel filtration buffer (2000 mM NaCl, 5 mM $MgCl_2$, 10% glycerol, 4 mM β-mercaptoethanol, 25 mM HEPES:KOH pH 8). The fractions containing RqcH-HTF were pooled and applied onto a HiPrep 10/26 desalting column (GE Healthcare) pre-equilibrated with storage buffer (720 mM KCl, 5 mM $MgCl_2$, 50 mM arginine, 50 mM glutamic acid, 10% glycerol, 4 mM β-mercaptoethanol, 25 mM HEPES:KOH pH 8). The fractions containing RqcH-HTF were collected and concentrated in an Amicon Ultra (Millipore) centrifugal filter device (cut-off 50 kDa). Protein preparations were aliquoted, frozen in liquid nitrogen.

B. subtilis *RqcP, wild-type* (VHp734, pET24d-rqcP-TEV-His_6) *and R16A variant* (VHp735, pET24d-rqcPR16A-TEV-His_6). C-terminally His_6-tagged protein was expressed in freshly transformed *E. coli* BL21 DE3 Rosetta (Novagen). Fresh transformants were inoculated to a final OD_{600} of 0.05 in the LB medium (800 mL) supplemented with 100 µg/mL kanamycin. The cultures were grown at 37 °C until an OD_{600} of 0.5, induced with 1 mM IPTG (final concentration) and grown for additional 1.5 hours at 30 °C. The cells were harvested by centrifugation and resuspended in binding buffer (500 mM NaCl, 5 mM $MgCl_2$, 20 mM imidazole, 10% glycerol, 1 mM β-mercaptoethanol, 25 mM HEPES:KOH pH 7.5) supplemented with 0.1 mM PMSF and 1 U/mL of DNase I. Cells were lysed by one passage through a high-pressure cell disrupter (Stansted Fluid Power, 150 MPa), cell debris was removed by centrifugation (25,000 rpm for 40 min, JA-25.50 Beckman Coulter rotor) and clarified lysate was taken for protein purification. Clarified cell lysate was filtered through a 0.2 µm syringe filter and loaded onto a 5 mL HisTrap HP column pre-equilibrated in binding buffer. The column was washed with 5 CV of wash buffer (2000

mM NaCl, 5 mM $MgCl_2$, 20 mM imidazole, 10% glycerol, 1 mM β-mercaptoethanol, 25 mM HEPES:KOH pH 7.5), and the protein was eluted with a linear gradient (6 CV, 0-100% elution buffer) of elution buffer (500 mM NaCl, 5 mM $MgCl_2$,500 mM imidazole, 10% glycerol, 4 mM β-mercaptoethanol, 25 mM HEPES:KOH pH7.5). Fractions most enriched in RqcP-TEV-His_6 (≈45-65% elution buffer) were pooled, totalling approximately 6 mL. The sample was diluted 3 times with dilution buffer (5 mM $MgCl_2$, 10% glycerol, 1 mM β-mercaptoethanol, 25 mM Tris-HCl pH 7.5) and loaded on HiPrep Q XL 16/10 column pre-equilibrated with binding buffer (150 mM NaCl, 5 mM $MgCl_2$, 10% glycerol, 1 mM β-mercaptoethanol, 25 mM Tris-HCl pH 7.5) and the protein was eluted with a linear gradient (3 CV, 0-100% elution buffer) of elution buffer (1000 mM NaCl, 5 mM $MgCl_2$, 10% glycerol, 1 mM β-mercaptoethanol, 25 mM Tris-HCl pH 7.5). Fractions most enriched in RqcP-TEV-His_6 were pooled, totalling approximately 12 mL, and applied on HiPrep 10/26 desalting column (GE Healthcare) pre-equilibrated with cleavage buffer (400 mM KCl, 5 mM $MgCl_2$, 10% glycerol, 4 mM β-mercaptoethanol, 25 mM HEPES:KOH pH 7.5). To cleave off the His_6 tag, 10 µg of His_6-Tev protease per 1 mg of RqcP were added and the reaction mixture was incubated at 10 °C for ≈14 hours. After the His_6 tag was cleaved off, the protein was passed through 5 mL HisTrap HP column pre-equilibrated with storage buffer (500 mM KCl, 5 mM $MgCl_2$, 10% glycerol, 1 mM β-mercaptoethanol, 25 mM HEPES:KOH pH 7.5). Fractions containing RqcP in the flow-through were collected and concentrated in an Amicon Ultra (Millipore) centrifugal filter device (cut-off 10 kDa). Protein preparations were aliquoted, frozen in liquid nitrogen.

Supplementary Table 1. Reference table for *B. subtilis* tRNAs array data.
tRNA^{Met}CAUi – initiator tRNA$_i^{Met}$ and tRNA^{Met}CAUe – elongator tRNA^{Met}.

Array probe designation	Recognised tRNA isoacceptor[a] (copy number)	Amino acid & codon(s) recognised by the tRNA	Anticodon of the tDNA array probes[b]
Ala-IGC	tRNA^{Ala}GGC (1)	Ala-GCC/U	A/GGC
Ala-A/C/UGC	tRNA^{Ala}UGC (3)	Ala-GCA/G/U	A/C/TGC
Arg-ICG	tRNA^{Arg}ACG (2)	Arg-CGC/U/A	T/A/GCG
Arg-C/UCG	tRNA^{Arg}CCG (1)	Arg-CGG	CCG
Arg-CCU	tRNA^{Arg}CCU (1)	Arg-AGG	CCT
Arg-UCU	tRNA^{Arg}UCU (2)	Arg-AGA	TCT
Asn-GUU	tRNA^{Asn}GUU (3)	Asn-AAC/U	A/GTT
Asp-GUC	tRNA^{Asp}GUC (4)	Asp-GAC/U	A/GTC
Cys-G/ACA	tRNA^{Cys}GCA (1)	Cys-UGC/U	A/GCA
Gln-C/UUG	tRNA^{Gln}UUG (4)	Gln-CAA/G	C/TTG
Glu-UUC	tRNA^{Glu}UUC (6)	Glu-GAA/G	C/TTC
Gly-GCC	tRNA^{Gly}GCC (2)	Gly-GGC/U	A/GCC
Gly-UCC	tRNA^{Gly}UCC (3)	Gly-GGA/G/U	A/C/TCC
His-GUG	tRNA^{His}GUG (2)	His-CAC/U	A/GTG
Ile-IAU	tRNA^{Ile}GAU (3)<br>tRNA^{Ile}CAU (1)	Ile-AUC/U<br>Ile-AUA	A/GAT<br>TAT
Leu-CAA	tRNA^{Leu}CAA (1)	UUG	CAA
Leu-CAG	tRNA^{Leu}CAG (1)	CUG	CAG
Leu-A/GAG	tRNA^{Leu}GAG (1)	CUC/U	A/GAG
Leu-UAA1	tRNA^{Leu}UAA (2)	UUA/G	C/TAA
Leu-UAA2	tRNA^{Leu}UAG (1)	CUA/G	C/TAG
Lys-UUU	tRNA^{Lys}UUU (3)	AAA/G	TTT
Met-CAU1	tRNA^{Met}CAUi (3)	Meti-AUG	CAT
Met-CAU2	tRNA^{Met}CAUe (1)	Met-AUG	CAT
Met-CAU3	tRNA^{Met}CAUe (1)	Met-AUG	CAT
Phe-GAA	tRNA^{Phe}GAA (3)	Phe-UUC/U	A/GAA
Pro-A/G/C/UGG	tRNA^{Pro}UGG (1)	Pro-CCA/G/U	A/C/TGG
Ser-GCU	tRNA^{Ser}GCU (2)	Ser-AGC/U	A/GCT
Ser-GGA	tRNA^{Ser}GGA (1)	Ser-UCC/U	A/GGA
Ser-A/C/UGA	tRNA^{Ser}UGA (2)	Ser-UCA/G/U	A/C/TGA
Thr-CGU<br>Thr-A/GGU<br>Thr-UGU	tRNA^{Thr}UGU (3)	Thr-ACG<br>Thr-ACC/U<br>Thr-ACA/G/U	CGT<br>A/GGT<br>A/C/TGT
Trp-CCA	tRNA^{Trp}CCA (1)	Trp-UGG	CCA
Tyr-GUA	tRNA^{Tyr}GUA (2)	Tyr-UAC/U	A/GTA
Val-mAC	tRNA^{Val}GAC (1)	Val-GUC/U	A/GAC
Val-UAC	tRNA^{Val}UAC (3)	Val-GUA/G/U	A/C/TAC

[a]Source: GtRNAdb (http://gtrnadb.ucsc.edu/) (4). Genomic copy number is included in parentheses.
[b]The anticodon designates the anticodon(s) implicated in the design of the complementary tDNA probes.

Supplementary Table 2. Strains and plasmids used in this work.

The table is provided as a separate Excel file.

Supplementary Table 3. Cryo-EM collection, refinement, and validation statistics.

	$RqcH^{DR}$-50S
**Data collection and processing**	
Magnification	165 000
Voltage (kV)	300
Electron exposure (e^{-}/Å^2)	34.8
Defocus range (μm)	−0.8–2.0.
Pixel size (Å)	0.82
Symmetry imposed	None
Initial particle images (no.)	145 631
Final particle images (no.)	16 700
Map resolution (Å)	3.2
FSC threshold	0.143
**Refinement**	
Map sharpening *B* factor (Å^2)	−75.814
Cross-correlation (volume)	0.80
Model composition	
Non-hydrogen atoms	93 862
Protein residues	3 818
RNA bases	2 996
R.m.s. deviations	
Bond lengths (Å)	0.011
Bond angles (°)	1.109
Validation	
MolProbity score	1.75
Clashscore	4.79
Poor rotamers (%)	0.23
Ramachandran plot	
Favored (%)	91.61
Allowed (%)	8.20
Disallowed (%)	0.19

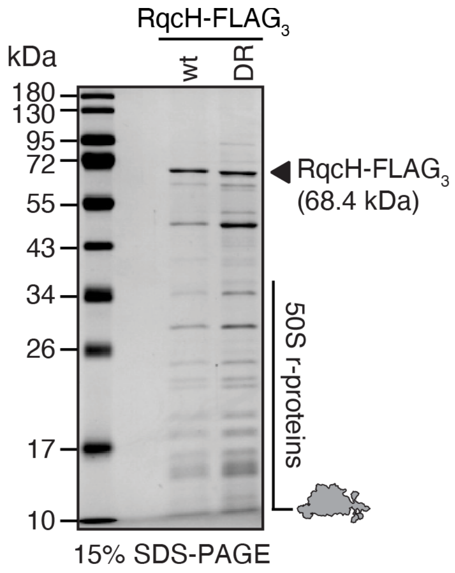

Supplementary Figure 1. Immunoprecipitation of RqcH^{DR}-FLAG$_3$-50S RQC complexes, related to Figure 4.

10 µL of co-IP samples eluted with poly-FLAG peptide and 10 µL of molecular marker were resolved on a 15% SDS-PAGE.

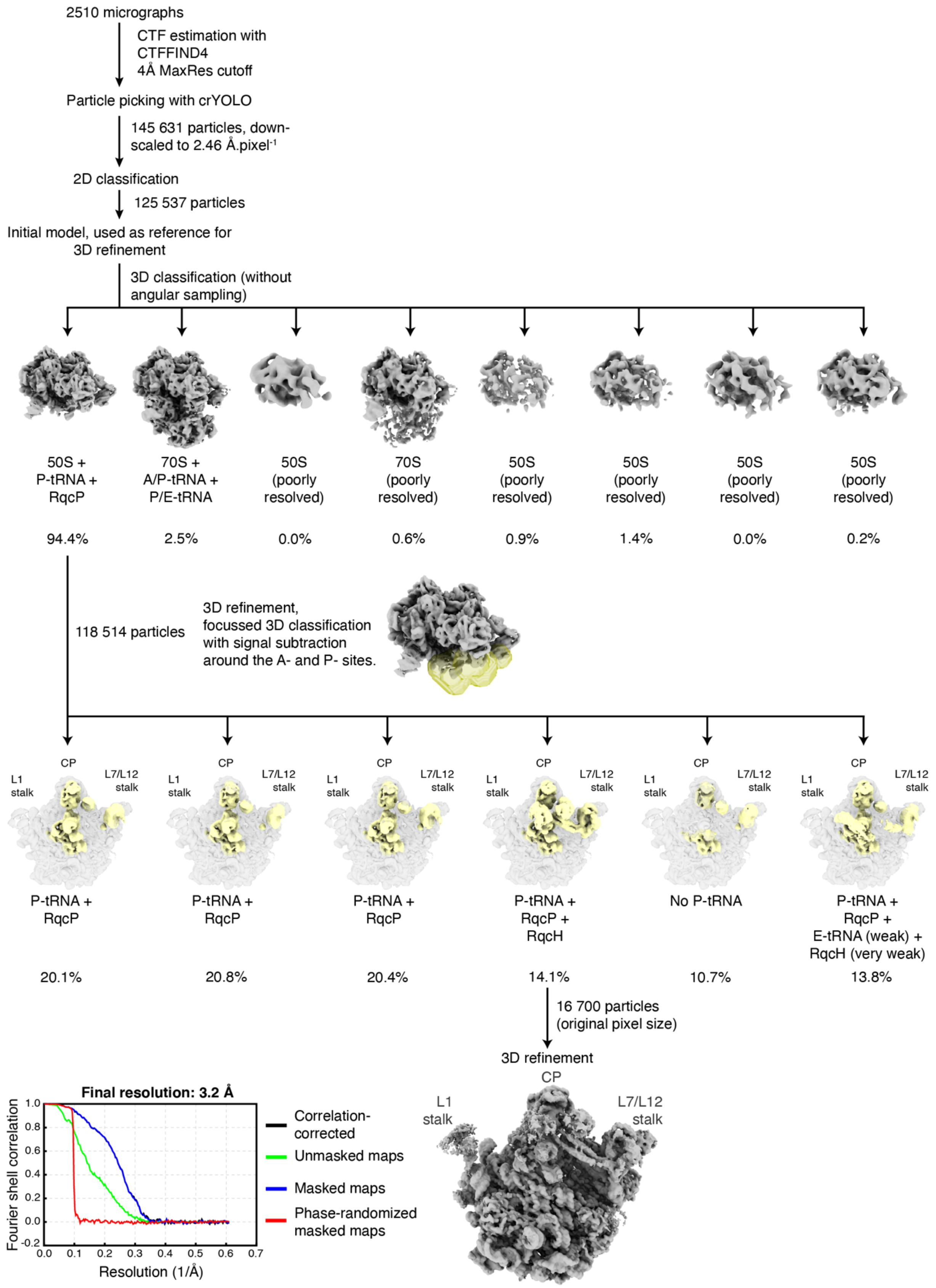

Supplementary Figure 2. Processing of cryo-EM data from RqcH^{DR}–FLAG$_3$ immunoprecipitation sample, related to Figure 4.
Processing scheme of RqcH^{DR} IP micrographs and FSC curve generated by RELION (5). Refer to methods for additional details.

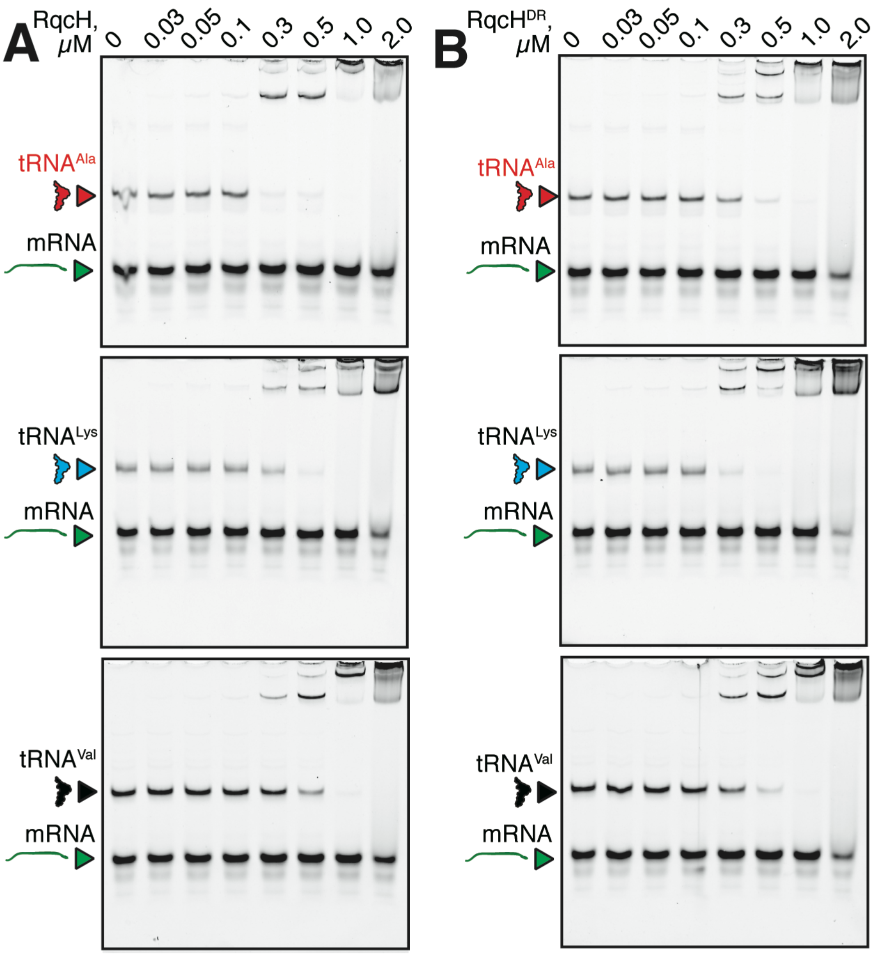

Supplementary Figure 3. Representative full-size EMSA gels, related to Figure 2.
Complex formation between 0.1 μM tRNA (either *E. coli* $tRNA^{Val}$ or *B. subtilis* $tRNA^{Lys}$ or *B. subtilis* $tRNA^{Ala}$) and increasing concentrations of *B. subtilis* RqcH-HTF (either wild-type or DR-substituted, D97A R98A) was monitored by EMSA. All experiments were performed in the presence of 1 μM of competing synthetic model mRNA(MVF) RNA oligonucleotide (5′-GGCAAGGAGGAGAUAAGAAUGGUUUUCUAAUA-3′). Gels were stained by SYBR Gold (Life Technologies) nucleic acid stain for 30 minutes.

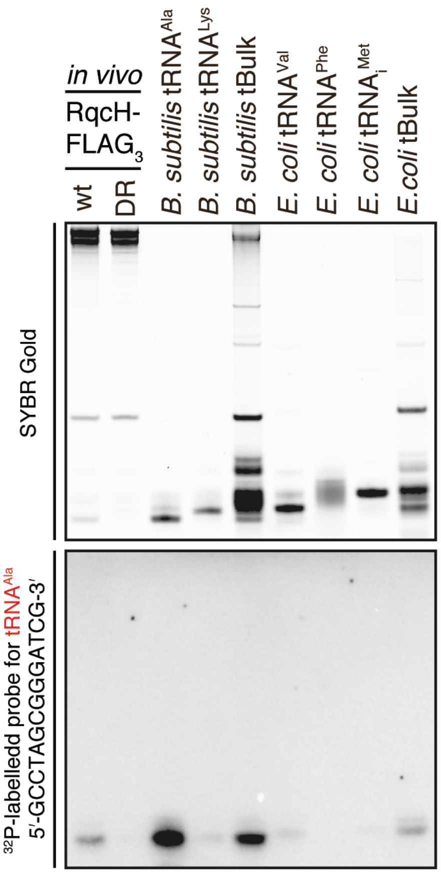

Supplementary Figure 4. Specificity of *B. subtilis* tRNA^{Ala} detection by Northern blotting, related to Figures 2 and 3.

RNA from co-IP samples (150 ng per lane), individual *B. subtilis* or *E. coli* tRNA species (1.5 pmol per lane) or either *B. subtilis* or *E. coli* total tRNA (9 pmol per lane) were resolved on 8M urea 8% polyacrylamide gels and either stained with SYBR Gold (Life Technologies) nucleic acid stain (top) and probed through Northern blotting using ^{32}P-labeled tRNA^{Ala} probe (bottom).

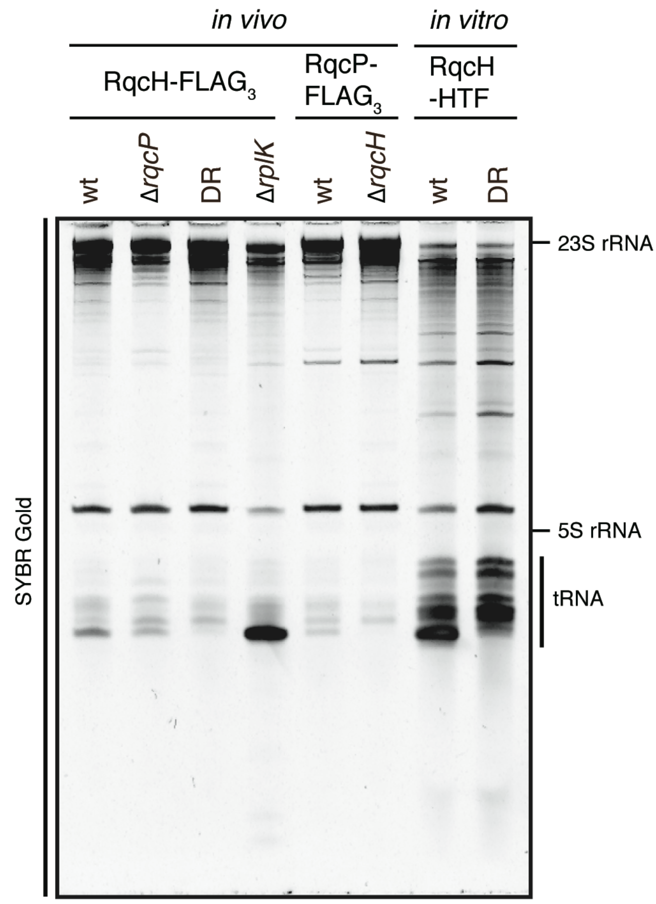

Supplementary Figure 5. SYBR Gold staining analysis of 50S RQC complexes isolated through co-IP of either RqcH-FLAG$_3$ or RqcP-FLAG$_3$ directly from lysed *B. subtilis*, related to Figures 3 and 5.
RNA from co-IP samples (150 ng per lane) were resolved on 8M urea 8% polyacrylamide gel and stained with SYBR Gold (Life Technologies) nucleic acid stain.

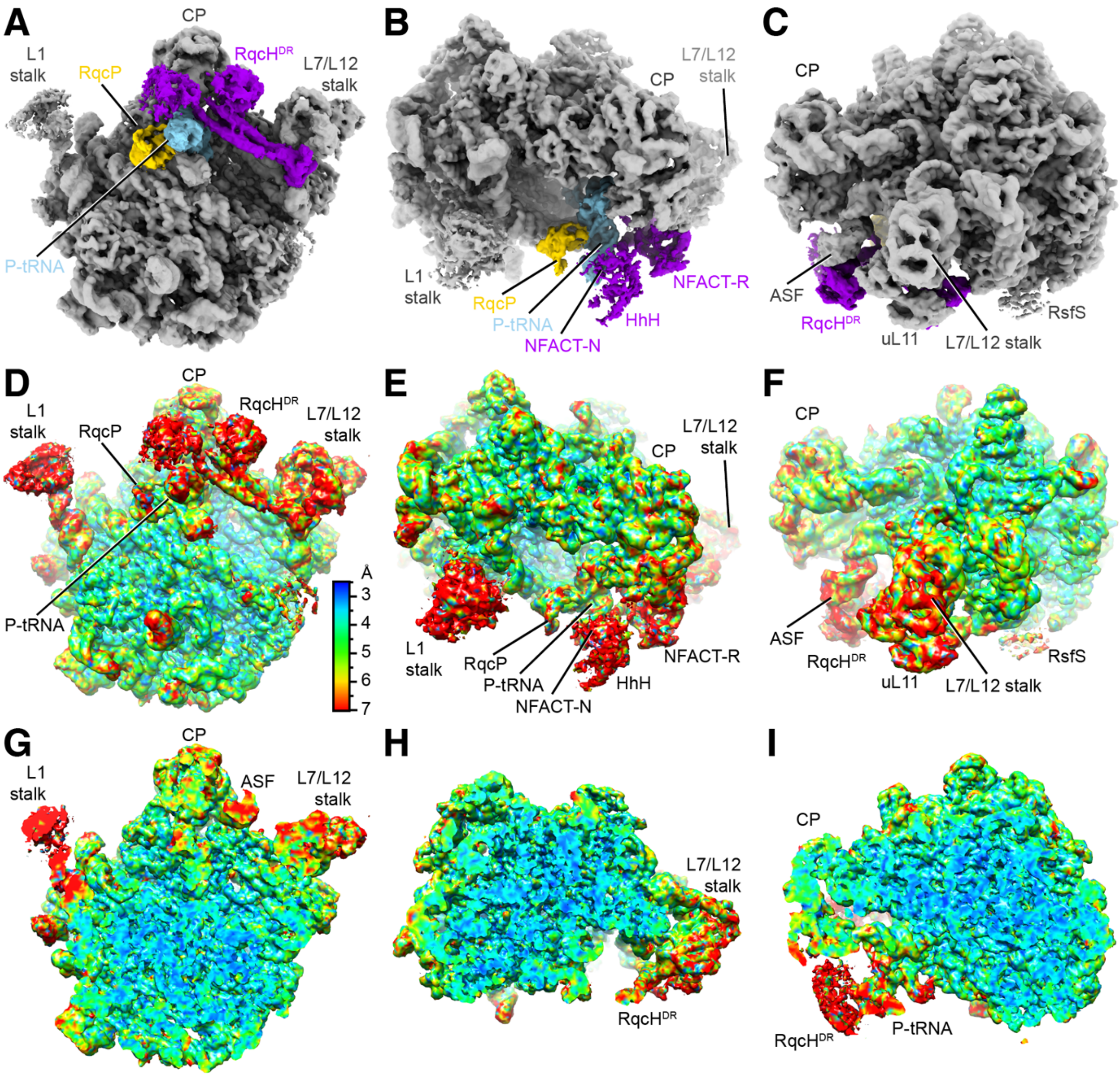

Supplementary Figure 6. Density features and local resolution of cryo-EM maps from RqcH^{DR}–FLAG$_3$ immunoprecipitation, related to Figure 4.
(**A**) Crown view of RqcH^{DR} bound to the 50S-P-tRNA-RqcP RQC complex. (**B, C**) Alternative views of **A**. (**D–I**) The same views as in **A–C** except coloured by local resolution. Either whole views (**D–F**) or cut-throughs (**G–I**) are shown.

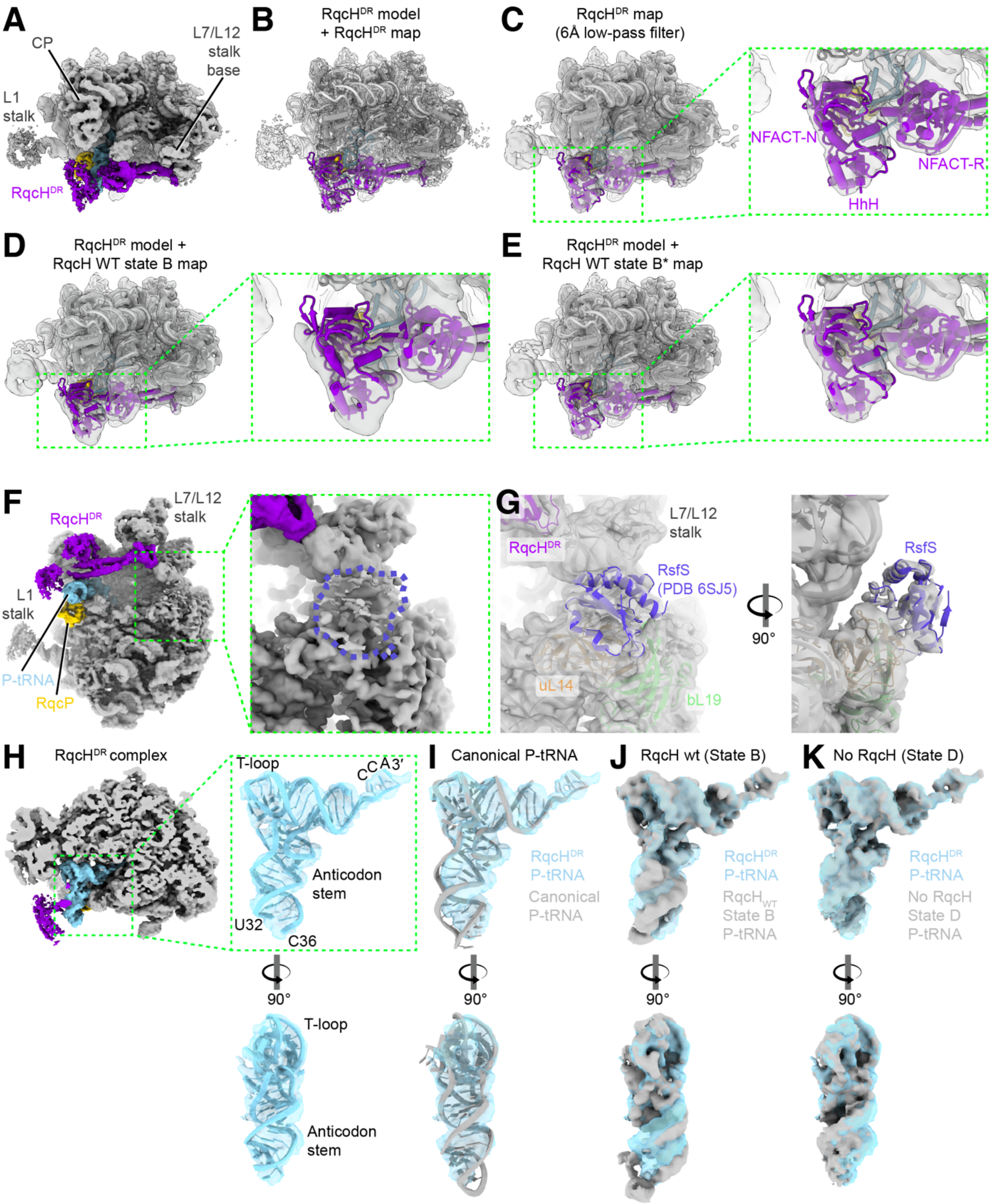

Supplementary Figure 7. Comparison of RqcH^{DR} and wild-type RqcH cryo-EM volumes, related to Figure 4.

(**A**) Overview of RqcH^{DR} bound to the 50S-P-tRNA-RqcP RQC complex. (**B**) as in A except fitted models are shown in a transparent map. (**C**) As in B, except with a 6 Å low-pass filtered map. Inset, right, is a close view of the RqcH globular domains. (**D**) The RqcH^{DR} model compared with the RqcH wild-type state B map low-pass filtered to 6 Å (EMD-11889). . (**E**) The RqcH^{DR} model compared to the wild-type RqcH state B* map low-pass filtered to 6 Å (EMD-11915). (**F**) Overview of the RqcH^{DR} complex with emphasis on the RsfS binding site (blue dashed outline in inset). (**G**) Two views of the crystal structure of *S. aureus* RsfS (PDB 6SJ5 (6)) fitted into the RqcH^{DR} complex map. (**H**) View of the P-tRNA bound to the RqcH^{DR} complex. Inset shows P-tRNA density and model. (**I–K**). Same as the inset in **H** except

RqcH^{DR}-bound tRNA (cyan density) is compared to either canonical P-tRNA (**I**, PDB 6CFJ (7)), state B tRNA from the wild-type RqcH structure (PDB 7AS8, **J**), or state D tRNA from a 50S–P-tRNA–RqcP complex but no RqcH (EMD-11914, **K**).

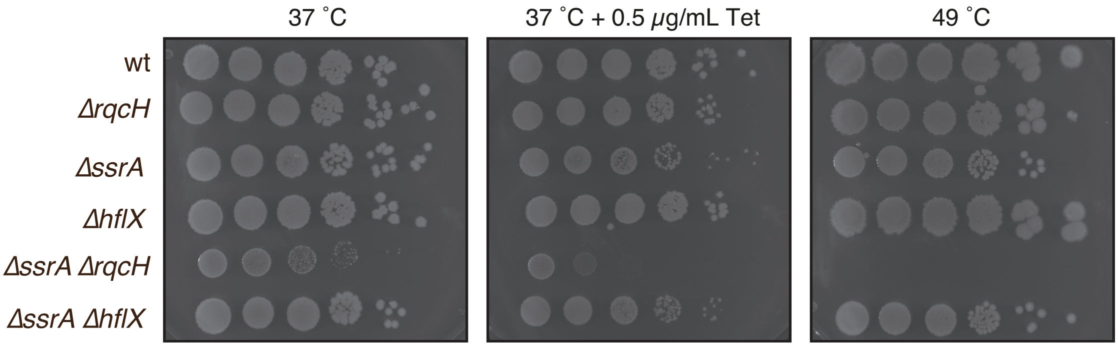

Supplementary Figure 8. HflX is does not play an essential role in RQC.
No synthetic growth defect was observed upon simultaneous *ssrA* and *hflX* deletion. The Δ*ssrA* Δ*rqcH* and Δ*ssrA* Δ*hflX* strains were used positive and specificity controls, respectively. 10-fold serial dilutions were spotted onto LB agar plates and incubated for 18 hours at 37 °C (left), 49 °C (right) or 37 °C in the presence 0.5 µg/mL tetracycline (Tet, middle).

Supplementary references:

1. Rezgui, V.A., Tyagi, K., Ranjan, N., Konevega, A.L., Mittelstaet, J., Rodnina, M.V., Peter, M. and Pedrioli, P.G. (2013) tRNA tKUUU, tQUUG, and tEUUC wobble position modifications fine-tune protein translation by promoting ribosome A-site binding. *Proc Natl Acad Sci U S A*, **110**, 12289-12294.
2. Murina, V., Kasari, M., Hauryliuk, V. and Atkinson, G.C. (2018) Antibiotic resistance ABCF proteins reset the peptidyl transferase centre of the ribosome to counter translational arrest. *Nucleic Acids Res*, **46**, 3753-3763.
3. Takada, H., Roghanian, M., Caballero-Montes, J., Van Nerom, K., Jimmy, S., Kudrin, P., Trebini, F., Murayama, R., Akanuma, G., Garcia-Pino, A. *et al.* (2021) Ribosome association primes the stringent factor Rel for tRNA-dependent locking in the A-site and activation of (p)ppGpp synthesis. *Nucleic Acids Res*, **49**, 444-457.
4. Chan, P.P. and Lowe, T.M. (2016) GtRNAdb 2.0: an expanded database of transfer RNA genes identified in complete and draft genomes. *Nucleic Acids Res*, **44**, D184-189.
5. Zivanov, J., Nakane, T., Forsberg, B.O., Kimanius, D., Hagen, W.J., Lindahl, E. and Scheres, S.H. (2018) New tools for automated high-resolution cryo-EM structure determination in RELION-3. *elife*, **7**, e42166.
6. Khusainov, I., Fatkhullin, B., Pellegrino, S., Bikmullin, A., Liu, W.T., Gabdulkhakov, A., Shebel, A.A., Golubev, A., Zeyer, D., Trachtmann, N. *et al.* (2020) Mechanism of ribosome shutdown by RsfS in *Staphylococcus aureus* revealed by integrative structural biology approach. *Nat Commun*, **11**, 1656.
7. Tereshchenkov, A.G., Dobosz-Bartoszek, M., Osterman, I.A., Marks, J., Sergeeva, V.A., Kasatsky, P., Komarova, E.S., Stavrianidi, A.N., Rodin, I.A., Konevega, A.L. *et al.* (2018) Binding and Action of Amino Acid Analogs of Chloramphenicol upon the Bacterial Ribosome. *J Mol Biol*, **430**, 842-852.
